# Be rich or don’t be sick: estimating Vietnamese patients’ risk of falling into destitution

**DOI:** 10.1186/s40064-015-1279-x

**Published:** 2015-09-21

**Authors:** Quan Hoang Vuong

**Affiliations:** Centre Emile Bernheim, Université Libre de Bruxelles, 50 Ave F.D. Roosevelt, Brussels, 1050 Belgium

**Keywords:** Health insurance, Government policy on health care, Risk of destitution, I13, I18, I19

## Abstract

This paper represents the first research attempt to estimate the probabilities of Vietnamese patients falling into destitution due to financial burdens occurring during a curative hospital stay. The study models risk against such factors as level of insurance coverage, residency status of patient, and cost of treatment, among others. The results show that very high probabilities of destitution, approximately 70 %, apply to a large group of patients, who are non-residents, poor and ineligible for significant insurance coverage. There is also a probability of 58 % that seriously ill low-income patients who face higher health care costs would quit their treatment. These facts put the Vietnamese government’s ambitious plan of increasing both universal coverage (UC) to 100 % of expenditure and the rate of UC beneficiaries to 100 %, to a serious test. The current study also raises issues of asymmetric information and alternative financing options for the poor, who are most exposed to risk of destitution following market-based health care reforms.

**Electronic supplementary material:**

The online version of this article (doi:10.1186/s40064-015-1279-x) contains supplementary material, which is available to authorized users.

## Background

Today, Vietnam has a population of 92 million, with a low per capita GDP of approximately $2000. Financial hardship is common among the populace, both in urban and rural areas. The poverty issue is much more serious with families who have a seriously sick member.

On November 13, 2014, an article on *Dan Tri*—a popular online media source in Vietnam—reported on a story about patient Nguyen Thi Lan, in Thach Lap Commune, Giong Rieng District, Kien Giang (a southern province of Vietnam). She suffered from a serious brain tumour that led to uncontrollable behaviour and unintentionally dropped her 1-year-old daughter five times. Her family could not afford travel and health care costs, so they kept her home and used “traditional medicine” without success. That article made numerous readers empathetic to her family’s plight; many sent money to help. On December 15, 2014, 47 million Vietnamese Dong (VND), approximately $2200, was collected from various readers and sent to her family, allowing Mrs. Lan to travel to a provincial hospital and start treatment (*Dan Tri Online*^2014^). Apart from showing public care about the hardship of their country fellows, this and similar articles also give rise to the issue of efficiency and use of health insurance, treatment costs and the general degree of financial destitution that many poor patients and their families face.

The amended Law on Health Insurance, effective January 2015, increases universal coverage (UC) to 100 % of the population, providing a full coverage of all relevant expenditures. The state expects that the new law will help reduce exposure of members of society, especially the poor, to the risk of destitution—which has for decades been a harsh reality—caused by extreme medical care costs that uninsured patients have little choice but to pay.

Unfortunately, the problem is hardly new. More than 13 years ago, Whitehead et al. (2001) discussed the problem of patients risking falling into ‘the medical poverty trap’, giving a ballpark figure: “In rural North Vietnam, 60 % of poor households were in debt, with a third citing payment for health care as the main reason”. Also, the authors called for researchers and policy-makers to pay attention to poverty-alleviation strategies, bearing the medical costs to vulnerable sections of society in mind (p. 834–6). This research has attracted considerable attention from the public and scholarly communities, leading to more articles addressing this issue in developing countries. The need for further microeconomic research on the household costs of illness and implications for poverty is imperative: “International research efforts also need to develop a common illness cost and impact methodology to allow more meaningful comparisons of the economic burden of illness across settings and diseases” (Russell 2004: p. 152).

While highlighting important role of health economic evaluation (HEE) in strategic planning and policy making, Tran et al. (2014) reviewed 26 HEE studies in Vietnam and call for connecting researchers and policy-makers. Their findings of limitation of scope and number of works as well as severe technical errors or omissions imply a need for more empirical studies to promote evidence-based policies. There are also encouragements to supply policy-making process with stylised facts. Jelicic Kadic et al. (2014)call for using high-quality evidence in Croatian health care policy to rationalize expenditures and to ensure wider and better access to medicines. Zhang et al. (2015) consider China’s National Reimbursement Ratio as a helpful quantitative indication in assessing and predicting national health insurance system. Santatiwongchai et al. (2015) affirm that there is room for improvement in the quality and usefulness of evidence to meet the need of governments and various development partners.

This article aims to identify factors that may affect the risk of destitution of Vietnamese inpatients based on a survey of patients who received inpatient hospital treatment. Many of the questions asked for perceptions of such critical determinants as severity of illness, distance of hospital from the patient’s home, and “thank-you money” for allegedly premium health care and treatment.

The article begins with a literature review on studies of Vietnam’s health care system, with an emphasis on insurance, costs and poverty. Next, it moves on to the research method of a baseline category logit model, which is employed to model the conditional probabilities of going destitute when certain specific events occur. The third section reports estimated results, together with computed probabilities, which address the research questions. The paper closes with a discussion of key insights and implications for patients, health service providers and the state.

## Literature review

Researchers have studied issues relating to health care systems, medical costs, the ‘poverty trap’, health reform policy-making and shed light on numerous aspects of low-income countries’ health care sector. This section briefly discusses issues related to the Vietnamese health care sector, which give rise to the research questions.

### Health care reforms and financing issues

Bloom (1997) sees the need for ‘radical health sector reforms’ for low-income countries, and states that China and Vietnam could exemplify a model of financing health services, especially in rural areas. However, both countries face the issue of rising health costs and inequalities among groups of different income levels. In the 1990s, a high proportion of rural people in Vietnam were able to consult with health workers in the community, and to Bloom: “This suggests that access to basic health services is reasonably good”. Still, understanding the impact of illness on risk of becoming financially distressed is more challenging due to scarcity of socioeconomic data. Quality of information for policy making is thus limited and seriously affected. In addition, development of financing mechanisms that assist in covering treatment costs has seen little progress and is still an issue for debate. Medical costs usually serve to be a ‘shock’ to household’s well-being.

Also in China, after a decade of reforms to significantly broaden government-backed insurance coverage and the availability of basic care, Daemmrich (2013, p. 1) notices the Chinese Government “are encountering a dilemma between supporting profit-seeking industries that offer the potential for new medical products and services but want free-market pricing, and public access to low-cost care that requires redistributive policies and price controls to function efficiently”. If one considers Vietnam’s reforms of health care system has started with amendment of the Law on Health Insurance and recent growth of private hospitals (Hort 2011) then overcoming such a dilemma is a challenge to the country’s policy makers. To this end, quantitative indications of financial matters—including probability of falling in destitution and factors that determine the probability—are helpful for both public and private players to do cost-benefit analysis.

Coping with rising medical costs, in either normal illness or a catastrophic event, means dealing with issues of increasing levels of debt and without understanding the probability of falling into a poverty trap, it will be hard to devise effective strategies for households to mitigate the risk of falling into financial hardship (Russell 2004: p. 153) as things have changed as the market modus operandi comes into play. Bloom (1997: p.16) provides some useful statistics: the richest quartile of rural Chinese spend 3.2 times as much on medical care as the poorest quartile; the figure for Vietnam was 4.6 times in 1994. Health care charges have become a burden for the poor, with rural Chinese spending up to five times the average daily per capita income on an average prescription. Vietnamese are spending 8 % of their annual non-food consumption for each visit to a commune health care station (Bloom 1997: p.16). The risk of falling into financial hardship jumps when there is a seriously ill family member, as average hospital admission could cost 60 % of the annual net income of poor households in China. Moreover, an average commune health unit admission costs 45 % of a poor family’s annual non-food consumption in Vietnam. An adverse health event can cause increasing debts and asset sales, and becomes an important cause of poverty. The poor have too few financing options. What is more, economic reforms have led to a situation in which the relationships between health workers, government and patients is altered, and health service providers now favour the rich, to whom they can supply expensive drugs and sophisticated technologies (Bloom 1997: pp. 17–18).

Regarding financing alternatives for the majority of patients, Sepehri et al. (2003) verify that Vietnam’s health care system has undergone major structural reforms, which significantly affect the delivery and financing of health services. Emerging issues are access, efficiency and equity in health services sector, and the trend of dwindling state funds and a shift from state financing to out-of-pocket fees paid by patients (p. 156). The rich tend to receive more health care, with longer hospital stays, and use more intensive resources than do the poor. The poor receive proportionally less care, with a rising trend of over-provision of services and expensive drugs, leading medical care costs to take up a larger percentage of a family overall income.

Specifically, Lönnroth et al. (2001) point to the fact that ‘evening clinic’—a kind of privately run health service operation used by out-patients—treatment of tuberculosis by private physicians may cost 200,000–1,000,000 Vietnamese Dong/month ($13–$67). For many households, that amount is a ‘heavy’ financial burden. Apart from fees and drugs, patients and household members were also worried about travel costs and time-consuming processes that usually triggered discontinued income during treatment periods, which could exceed fees and the costs of drugs (Lönnroth et al. 2001: p. 940–3).

In a broad and highly influential study, Whitehead et al. (2001) unveil that poor households reporting illness in a rural area in northern Vietnam spent on average 22 % of their household budget on health-care costs, whereas rich households spent 8 % (p. 834). In this report, the authors do not state explicitly the definition of ‘rich’ and ‘poor’ patients and rather refer to the World Bank’s classification. That is why ‘home remedies’ are still a preferred choice among the poor, representing ‘the cheapest healthcare option’ although the average cost rose progressively due to the price of drugs and consultations (Segall et al. 2002: p. 500). While Segall et al. (2002) note that non-poor households spent on average 150 % of their monthly income, the lowest cost by the poor represented 200 % of their monthly income. Nonetheless, due to the income gap between the two groups, on average, non-poor households spent much more than the poor per admission in value. In rural areas of Vietnam, 3.3–10 % of the annual income per capita was devoted to health care—while an average of 2–7 % was typical in a variety of developing countries—leading many Vietnamese households to also sell rice reserves and livestock, apart from borrowings, to finance health costs (Segall et al. 2002: pp. 501–2). Therefore debt, as a major financing option for healthcare services, remained pervasive among the poor.

In the same vein, Ha et al. (2002) confirm the burden on households in rural areas and report that severely ill people tend to use public care (p. 61), although public services showed a tendency to consume more resources than private services, that in part means these services tend to cost more. The authors estimate that the amount of subsidy was quite small, in fact negligible, accounting for around 4 % the of total expenditures (pp. 67–8). Also, new issues emerge to exacerbate the problem of the financial burdens of health care, as Ensor (2004: p. 245) adds, “there is growing evidence to suggest that unofficial health care fees are likely to distort health care priorities and change the impact of health system reform” in developing countries. This also applies to the situation of Vietnamese health care sector as confirmed by results reported by Nguyen et al. (2012) upon surveying 706 households in 2008.

As to factors giving rise to the risk of poverty, Sepehri et al. (2005) postulate a possible link between income and length of hospital stay, as in transition economies post-hospital follow-up is virtually non-existent and travel is costly. According to the authors, a longer stay may increase assurance, reduce post-treatment complications and readmission, or simply speaking: better-quality care (p. 97). They suggest further investigations to examine the effects of unofficial and official payments on the intensity and quality of health care (p. 98) and the differences between groups of patients. This postulation by Sepehri et al. (2005) appears to be relevant to observations of Vietnamese patients and worth looking at. On the one hand, due to inadequate facilities some upper-tier hospitals such as Viet Duc have the policy of providing intensive care for most cases so that the length of stay for in-patients reduces to 7 days, whenever possible. One the other hand, there is certainly evidence of unnecessary in-patient care and excessive length of stay encouraged by other hospitals, aimed at higher average revenue collected per patient. There is no significant difference between health care fees between the poor and non-poor in public health services; it is likely that public sources may subsidise the rich rather than the poor (Thuan et al. 2008: p. 7). In light of this, Ekman et al. (2008: p. 252) conclude that there is an imperative need for reforming Vietnamese health insurance to focus on: (1) sustained resource mobilisation; (2) comprehensive functions of the health financing system; and (3) a long-term view of health insurance reform. Although roughly 50 % of the population benefit from some form of health insurance, only 18 % of the poor are entitled to these limited benefits, mainly channelled through the so-called Health Care Funds for the Poor (HCFP); 3/4 of which come from the central government and 1/4 come from a provincial source (Ekman et al. 2008: p. 255). The reality is that voluntary health insurance is still not easy and exhibits the asymmetric information issue.

What we learn from the extant literature is that although market reforms improve availability of health services, financing issues have arisen due to the tendency of inflating health care costs, in many cases unnecessarily. Debt financing for seeking health services has been common, especially among the poor, which subsequently increases the possibility of going destitute.

In addition, while emphasizing financial burden of medical care on the poor (Sepehri et al. 2003, 2005; Segall et al. 2002), especially patients who come from rural areas (Bloom 1997; Whitehead et al. 2001; Ha et al. 2002; Nguyen et al. 2012), the authors suggest distance from patient’s home to treatment facilities matters. Lönnroth et al. (2001: p. 940), indeed, take a note on the cost of travel.

In developing economies, trying to access to urban health care services is a common practice of rural patients. Bronstein and Morrisey’s work (1991) on data from 1983 and 1988 on hospital use in Alabama (USA) provides empirical evidence for increasing proportion of rural pregnant women travelling to metropolitan areas for infant services. Parkhurst and Ssengooba (2009) tell the same story in Uganda. Buczko (1994) affirms that rural hospitals are often bypassed by aged patients. The reasons may include avoiding assumingly inadequate care and accessing to advanced medical procedures. Moreover, Paul (1999) reports on widespread incidence of national health care bypassing in Bangladesh. Bangladeshi patients prefer foreign health care services because of lower costs, availability of specialized care, and better quality of services. Leonard et al. (2002) also consider strong preference of quality as a major reason for bypassing in Tanzania.

In Vietnam, the enforcement of amended Law on Health Insurance commencing on 1 January 2015 makes bypassing a burning issue. Although the Vietnam Ministry of Health unveils that 70 per cent of bypassed treatments are unnecessary (Nam Phuong 2015) and bypassed patients are eligible for much lower insurance payment [in comparison to previous regulation] the amendment is reportedly fail to prevent bypassing. Hospitals in economic hub Ho Chi Minh city reported a surge of patients declaring “non-insurance”. Oncology Hospital in the city noticed the number of non-insurance patients went up by 250 per cent. Many insured patients decide to declare uninsured since the insurance payment is so little in comparison to other expenses such as travelling and accommodation for family members who escort the patients during treatment period], a representative of the Hospital told *Tien Phong Newspaper* (Quoc Ngoc 2015).

### Use of health services, costs and insurance benefits, and treatment outcome

As health sector reform takes place, user fees grow. A major problem with user fees is that, although they help relieve the financial burden on the government, these fees can drive people into poverty and widen the gap between the rich and the poor. The need to establish measures for protecting the poor is imperative, especially in eliminating unofficial payments and asymmetric information between providers and patients. While only a small proportion of rural residents are eligible to receive health insurance benefits, low insurance coverage also increases the burden on the poor (Dao et al. 2008: pp. 1076–7).

Another issue is that statistics may have been biased due to the finding that the poor are likely to “modify the perception of sickness” to avoid costs due to health care needs and discontinued income (Thuan et al. 2008: p. 5). The poor show a higher tendency of using self-treatment, while the expenditure for self-treatment is only 13 % of the total curative expenditure. A possible explanation of this low expenditure ratio is because actual self-treatment costs tend to be under-reported.

Regarding health insurance, Liu et al. (2012) report significant differences in health insurance coverage between Vietnam and China (employing a data set containing observations from two provinces at different levels of economic development, Shandong and Ningxia) although the two countries share similar systems and socio-economic properties. Through a survey of six counties in China, the authors reported coverage rates ranging from 85 to 91 %, but the rate is much lower in Vietnam, which is about 50 %, including both voluntary and compulsory schemes. Still, while insurance coverage levels may be high in rural China, the benefit package is limited and co-payment ratio is high, disadvantaging the poor. Dang et al. (2006) offered a detailed comparison between the Chinese and Vietnamese. Vietnamese patients with health insurance are significantly more likely than uninsured to utilise in-patient services (Liu et al. 2012: p. 5). Vietnamese perceive that the insured receive poorer quality of services than non-members, reflecting their complaints that using insurance leads to prescription of only limited types and amounts of medicine and longer waiting time. Thus, it is quite common that insured patients go to private drug sellers for medicines that are ineligible under the public scheme (Liu et al. 2012: p. 6). With respect to the common practice of using private healthcare providers, a ready explanation is because patients are not seriously ill and therefore do not require complicated process of treatment. However, there are other factors also taken into account in making such decisions: (a) inadequate understanding of the risk of inappropriate treatment; (b) convenience for patients’ relatives; and, (c) trust on ‘rumors’ about reputation and efficacy of treatment methods by some local physicians, especially in rural areas where the use of traditional medicines (including herbal medicines) is common.

The relationship among the variables of use of health services, costs and insurance coverage is anticipated. Nonetheless, the impact of these factors, specifically costs and insurance coverage, on the treatment outcome is not obvious partly because they depend on the criticality of the patients when hospitalized. Thus, it is difficult to generalize the relationship, and there is little discussion on this specific issue.

### ‘Sensitive’ issues relating to out of pocket (OOP) payment

Regarding financing mechanisms in developing countries, the ‘implied’ risk of inflating the financial burden has become clearer with unreported out-of-pocket (OOP) payments by patients. Van Doorslaer et al. (2006) surveyed eleven low income countries and found that in Vietnam (as well as Bangladesh, China, India, and Nepal), more than 60 % of health care costs are paid out-of-pocket, and OOP health payments exacerbate poverty (p. 1357). Moreover, 2–7 % of the population in the eleven countries may fall below the extreme poverty threshold ($1/day) due to health care payments. The authors also suggest country policy makers conduct evaluations to learn more about specific reforms in health financing that could help reduce impoverishment due to health care payments (pp. 1362–1364).

Again, Van Doorslaer et al. (2007) found that the OOP share remains highest in Bangladesh, India and Vietnam, with 10.6–12.6 % of non-food expenditures spent on health care (p. 1169). These same three economies also continue to have the highest incidence of catastrophic payments (p. 1173). Chaudhuri and Roy’s (2008: pp. 42–44) report that OOP payment is positively related to per capita consumption, and increases for higher consumption quintile, revealing differences in the redistributive effect, the additional costs due to OOP payment would likely deter the Vietnamese poor from seeking health services.

In countries with such high levels of catastrophic healthcare expenditure and significant OOP payment, Xu et al. (2007) suggest a need to move away from OOP payments, using prepayment systems, ‘financial risk protection strategies’, and increasing funds for alleviating social inequalities in health care (pp. 981–982). In India, Karan et al. (2014) report that financial burden of OOP spending increases faster among disadvantaged groups, in comparison to the more advantaged or wealthy.

In Vietnam, the OOP issue has become even more ‘sensitive’ as more retired state employees are affected. They had used the state-subsidized healthcare system and been covered almost fully. For the rest of the society, the OOP payment requires paying bribes to doctors, nurses and hospital staffs in hopes for better care. In fact, Vietnamese patients tend to regard the OOP to cover extra medicine as the ‘new normal’ but remain highly uncomfortable with OOP ‘envelops’, although this practice has become widespread. The issue has been regarded as ‘sensitive’ (everybody knows but nobody tells) in transition economies like Vietnam and China, where health care infrastructures are inadequate and underinvested, and generally inefficient. The issue of “thank you money” as part of the expected OOP payments can become highly political, too.

The literature review suggests that researchers agree on: (1) the need for alternative financing for patients in developing countries, in particular Vietnam, and especially for the poor; (2) the implied risk of falling into destitution is high, especially for the poor; (3) there is a pressing need to better understand the relationships between socio-economic factors that help explain financial distress faced by the poor; and, (4) there is inadequate protection, at least via the health insurance system, for the poor. This suggests the need for empirical investigations to examine financing issues, illness, insurance, end result of treatment, health care costs, length of stay, ‘envelope OOP’ and the probability of post-treatment destitution, for different groups of patients. Although not all factors will have simultaneous or equal effects on the post-treatment financial conditions and treatment result, the research suggests likely relationships among several. That is what this study sought to explore.

## Research questions and method

Although the existing research significantly contributed to the understanding of the Vietnamese health care systems and issues with patients’ hardship, there is little about the probability of patients falling into destitution. In addition, little research examines the factors that enhance risk to patients when they have to decide whether to use health care services. Such insights could inform the policy making process in Vietnam by identifying critical factors and directions for improvements.

### Research questions

Improving the understanding of the Vietnamese health sector and patients’ risks involves answering the following research questions (RQ), which would complement existing knowledge and may contribute to upcoming health sector reform:

RQ1: Does residency status of patients and insurance coverage determine the probability of patients falling into indebtedness? The specific factor of residency status is important in Vietnam because society has for long been skeptical about provincial healthcare, leading patients to travel to major urban hospitals in Hanoi, Hai Phong, or HCMC. Doing so involves the travel costs, care taking that family members must provide and informational asymmetry about drug prices, treatment schedules, the best hospital to visit and even ‘right amount’ of “extra thank-you money” OOP.

RQ2: As for two most important factors to Vietnamese patients/households, i.e. treatment costs and illness, is there evidence to support this view and if yes, whose influence better explains the possibility of end results of treatment, empirically?

RQ3: Can the likelihood of paying too little or too much out-of-pocket “extra thank-you money” be determined by the severity of illness and/or income of patients? This OOP amount may be significant but if a patient appreciates the value of service, he/she would be willing to pay depending on his/her availability of finance, before or after the course of treatment.

### Research method

The multi-category logit models (also known as, polytomous logistic regression analysis) will be used to investigate the RQ1–3; the resulting models show behaviours of multinomial response variable (*Y*) following multinomial (and binomial) predictor variables.

The specific analysis employed in this article is baseline-category logits (BCL). This type of modelling enables us to detect relationships between discrete variables, and in this kind of survey, likely polytomous response variables and discrete (multinomial or binomial) explanatory variables. In addition, it allows us to compute useful probabilities upon specific events of hypothetical influence.

Although log-linear models are also useful in modelling this type of problem, logistic regression is preferred due to: (1) fewer and thus more significant variables and (2) direct interpretation of the estimated coefficients in measuring the empirical probabilities of events. Moreover, BCL models provide a simultaneous representation of the odds of being in one category relative to being in a designated category, called the baseline category, for all pairs of categories.

In this investigation, a patient (among *n* patients) can be regarded as independent and identical, and may have outcome in any of *J* categories for each factor to be investigated. Let $y_{ij} = 1$ if patient *i* has outcome in category *j* and $y_{ij} = 0$ otherwise. Then, ${\mathbf{y}}_{ij} = (y_{i1} ,y_{i2} , \ldots ,y_{ic} )$ represents a multinomial trial, with $\sum_{j} y_{ij} = 1$. Denote $n_{j} = \sum_{j} y_{ij}$ the number of “trials” having outcome in category *j*, the count $(n_{1} ,n_{2} , \ldots ,n_{c} )$ have a multinomial distribution. Let $\pi_{j} = P(Y_{ij} = 1)$ denote the probability of outcome in category *j* or each patient, then the multinomial probability mass function is computed as follows:

$$ p(n_{1} ,n_{2} , \ldots ,n_{c} ) = \left( {\frac{n!}{{n_{1} !n_{2} ! \cdots n_{c} !}}} \right)\pi_{1}^{{n_{1} }} \pi_{2}^{{n_{2} }} \cdots \pi_{c}^{{n_{c} }} . $$

This distribution has the following properties:

$$\begin{array}{*{20}c} {{\text{E(}}n_{j} )} & = & {n\pi_{j} } \\ {{\text{var(}}n_{j} )} & = & {n\pi_{j} (1 - \pi_{j} )} \\ {{\text{cov(}}n_{j} ,n_{k} )} & = & { - n\pi_{j} \pi_{k} }. \\ \end{array} $$

where $$ \sum_{j} n_{j} = n $$. 

Now, let $\pi_{j} ({\mathbf{x}}) = P(Y = j|{\mathbf{x}})$ represent a fixed setting for predictor variables, with $\sum_{j} \pi_{j} ({\mathbf{x}}) = 1$. Count data are grouped into *J* categories of *Y* as multinomial with corresponding sets of probabilities $\{ \pi_{1} ({\mathbf{x}}), \ldots ,\pi_{j} ({\mathbf{x}})\}$.

The baseline category logit models align each response (dependent) variable with a baseline category, taking the form:

$$ { \ln }\frac{{\pi_{j} ({\mathbf{x}})}}{{\pi_{J} ({\mathbf{x}})}} = \alpha_{j} + {\varvec{\upbeta}}_{j}^{\prime} {\mathbf{x}}, \quad j = 1, \ldots ,J - 1. $$

BCL analysis simultaneously models the effects of **x** on $(J - 1)$ logits, which in general vary according to the response paired with the baseline category. The estimating of $(J - 1)$ equations employing a given empirical data set would provide for parameters for these logits, as:

$$ { \ln }\frac{{\pi_{a} ({\mathbf{x}})}}{{\pi_{b} ({\mathbf{x}})}} = { \ln }\frac{{\pi_{a} ({\mathbf{x}})}}{{\pi_{J} ({\mathbf{x}})}} - { \ln }\frac{{\pi_{b} ({\mathbf{x}})}}{{\pi_{J} ({\mathbf{x}})}}. $$

The empirical data set, which contains count data and mainly uses categorical variables, would enable the computing of Pearson-type likelihood ratio test statistics ($X^{2} ,G^{2}$) or goodness-of-fit.

The polytomous logistic model is estimated as a multivariate generalized linear model (GLM) which takes the form:

$$ {\mathbf{g}}({\varvec{\upmu}}_{i} ) = {\mathbf{X}}_{i} {\varvec{\upbeta}}, $$

where, ${\varvec{\upmu}}_{i} = {\text{E(}}{\mathbf{Y}}_{i} )$, corresponding to ${\mathbf{y}}_{i} = (y_{i1} ,y_{i2} , \ldots )^{{\prime }}$; row *h* of the model matrix ${\mathbf{X}}_{i}$ for observation *i* contains values of independent variables for $y_{ih}$. For a BCL model, ${\mathbf{y}}_{i} = (y_{i1} ,y_{i2} , \ldots ,y_{i,J - 1} )^{\prime}$; thus $y_{iJ}$ is redundant. Therefore, for BCL:${\varvec{\upmu}}_{i} = (\pi_{1} ({\mathbf{x}}_{i} ),\pi_{2} ({\mathbf{x}}_{i} ), \ldots ,\pi_{J - 1} ({\mathbf{x}}_{i} ))^{'}$.and,

$$ g_{j} ({\varvec{\upmu}}_{i} ) = {\text{ln\{ }}\mu_{ij} /[1 - (\mu_{1} + \cdots + \mu_{i,J - 1} )]{\text{\} }}. $$

A rich account of technical details for practical modeling of polytomous logistic models is provided in Agresti (2002: pp. 267–74). Actual estimations performed in this study—whose results are reported in the next sections—employ analysis in R, following a set of instructions provided by Penn State at https://onlinecourses.science.psu.edu/stat504/node/171.

As a main purpose of the estimation is to compute response probabilities from multinomial logits, i.e. $\{ \pi_{j} ({\mathbf{x}})\}$, the following computation will apply:

$$ \pi_{j} ({\mathbf{x}}) = \frac{{{\text{exp(}}\alpha_{j} + {\varvec{\upbeta}}_{j}^{'} {\mathbf{x}} )}}{{1 + \sum\nolimits_{h = 1}^{J - 1} {{ \exp }\left( {\alpha_{h} + {\varvec{\upbeta}}_{h}^{'} {\mathbf{x}}} \right)} }}. $$

with $\sum_{j} \pi_{j} ({\mathbf{x}}) = 1$; $\alpha_{J} = 0$ and ${\varvec{\upbeta}}_{J} = 0$. The computed probabilities can be used to model the risk of a patient to fall into a category of financial distress (indebtedness or destitution) conditional upon some other “events” such as “being in the lower socio-economic status group” (SES) and/or “being non-resident” as to where the hospital is located, and/or “being insured”, and so on.

## The data set and estimations

### The survey, data and description

The survey was conducted by a team including hospital personnel and a Hanoi-based research firm, collecting data from inpatients of many hospitals in northern Vietnam including but not limited to: Viet Duc Hospital, Bach Mai Hospital, Vietnam-Japan Hospital, Hai Duong Polyclinic Hospital, Thai Binh Polyclinic Hospital, Ministry of Transports Polyclinic, to name just a few.

Interviewers approached patients individually and gradually acquired information for the survey, including questions about “sensitive data” that a more general/social survey could hardly obtain. Such questions included family status, patient’s income level, patient’s extra expenses to doctors and hospital’s staff, and their borrowings money to finance treatment (Additional file 1).

The research team obtained qualified data for 330 patients, from a total of approximately 1000. The data team consists of six people, one in charge of coordinating and checking quality, two in putting data into the database, and three of data collecting from hospital sources. These 1000 interviewees were selected randomly from the hospital records and based on the judgement by data collecting people about whether the patient/relative is available and/or willing to participate, after explaining about the ethical standards, issues of information nondisclosure and the possible insights the survey may contribute to the understanding of policy-makers and public in general. Sometimes a respondent had been approached multiple times over few weeks before he/she agreed to answer the survey completely. Nearly 400 participated and only 330 were considered of satisfactory quality for the subsequent analysis. The survey process started in first week of August 10, 2014 and ended first week of February 2015 (Additional file 2).

The following variables directly or indirectly enter into the analysis process:

- “Res”: if a patient is considered to be a “resident” of the region where a hospital is located;
- “Stay” if a patient’s stay in the hospital is less than 10 days (‘short’) or equal-or-greater-than 10 days (‘long’)
- “Insured” if a patient is entitled to some insurance coverage under the UC or specific coverage provided by an employer;
- “SES” had four levels of socioeconomic status for the patient/household: very high/rich; high; medium; low;
- “Illness” representing the severity of sickness of the patient when hospitalised
- “IncRank” showing the income level of a patient;
- “Spent” and “Dcost” represent amounts spent during the treatment period and average daily cost paid by the patient, in millions of Vietnamese Dong (VND 1 million = $47.2);
- “Pins; Pinc; Pchar; Ploan”: percentage of payment by sources of insurance coverage, savings, charity funds, and borrowings, respectively;
- “Streat, Srel, Senv”: percentage of spending on direct treatment costs, relatives and friends for caring for the patient, “thank-you envelops”, and OOP, respectively;
- “Burden”: levels of financial burden on the patient/household following treatment; and,
- “End”: end outcome of treatment telling if the patient fully recovered, partially recovered, stopped treatment in the middle of the process, or stopped treatment earlier due to lack of financing options.

Detailed information for all variables and their categorical values are provided in “Appendix 1''.

**Fig. 1:**
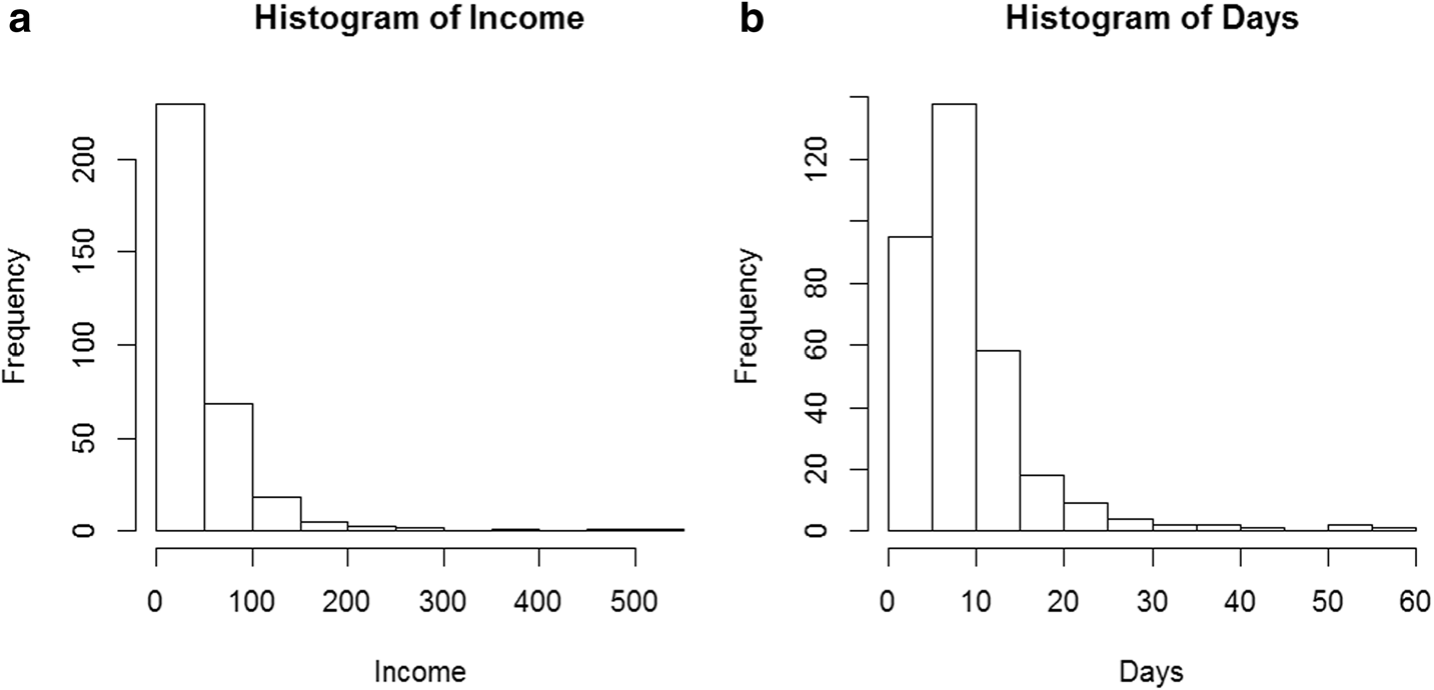
Frequency distribution of patients’ age and time spent in the hospital. **a**–**b** Two histograms for patients survey with respect to income levels and corresponding stays in hospital

An empirical distribution of income and hospital stay among patients constituting the sample is shown in Fig. 1. In the dataset, these three factors are represented by the quantitative variables Age, Days and Income [in millions of Vietnamese Dong (VND) per year]. A large portion of the sample is constituted by inpatients that stayed less than 10 days in the hospital. In addition, a large portion of patients have incomes lower than VND 50 million (approximately $2360) per year, and patients with annual incomes below $4720 account for more than 90 % of the sample (see Fig. 1a). Likewise, the majority of patients stay less than 10 days in the hospital (Fig. 1b).

**Fig. 2:**
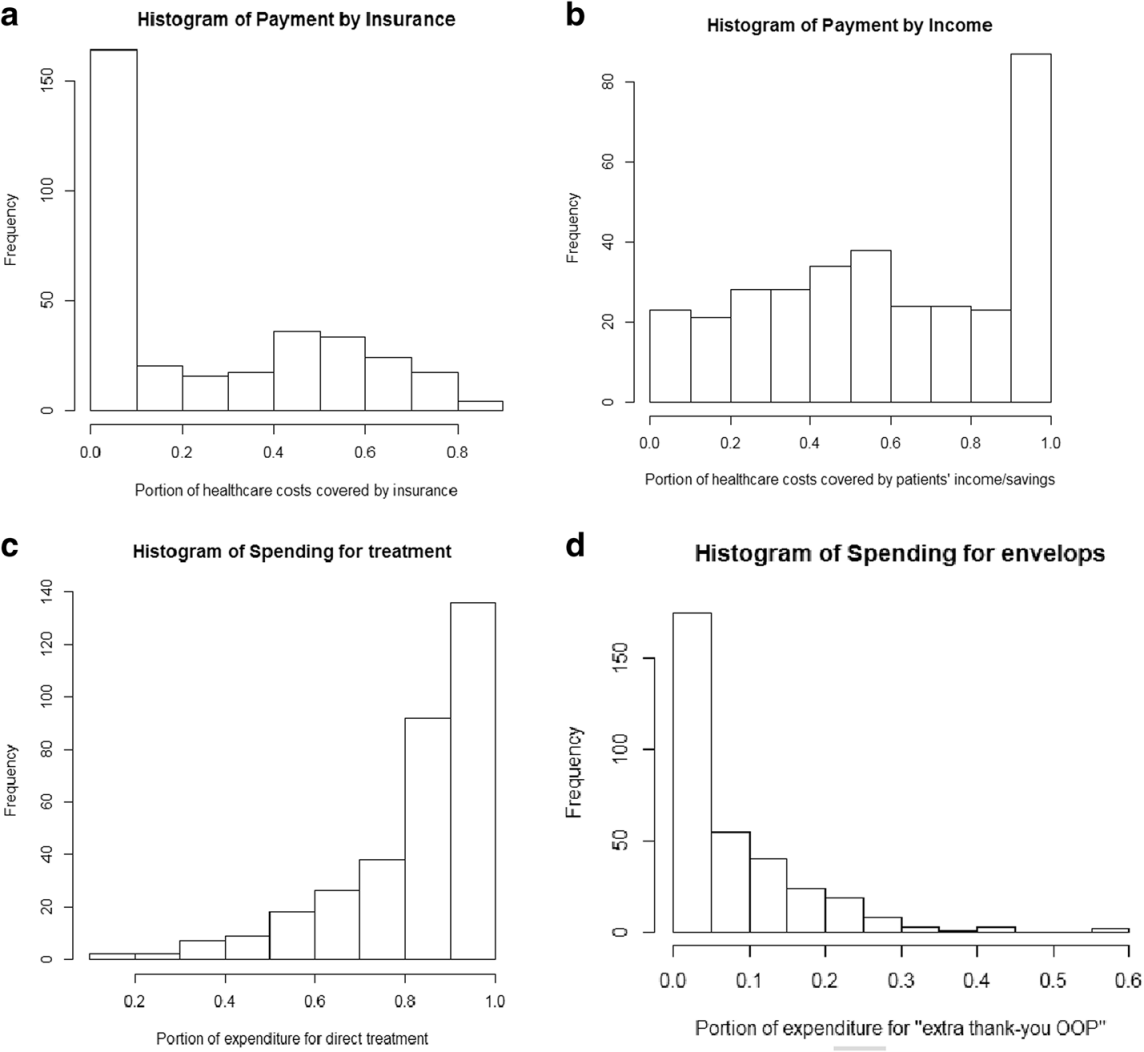
Sources of financing and cost structure for patients. Four histograms for related factors values learned from the survey. Specifically, **a** and **b** shows histograms for ratio of financing from insurance and income/savings respectively, while **c** and **d** refer to the propensity of use of funds for purposes of treatment versus of “building relationship” with doctors and hospital staffers

Figure 2 presents sources of financing for paying health care costs by patients, from insurance policy reimbursement (Pins) to savings from the patients and their family members (Pinc). These are also quantitative variables measured in percentages. Clearly, a majority of surveyed patients receive less than 50 % reimbursement from their insurance coverage; income/savings represents the single most important financing source for paying health care costs for the majority of patients (Fig. 2a, b).

Likewise, the “Histogram of spending for treatment” in Fig. 2 shows the frequency of patients paying for the main costs of treatment (e.g., hospital room, medicines, use of equipment, nurse care); “Spending for envelops” is the portion of a patient’s total payments for extra costs to doctors and hospital’s staff in the popular form of “envelop” (thank-you money and/or bribe). It can be seen that the majority (80–100 %), of patients’ expenses are for direct treatment costs and hospital services, while the majority of patients pay less than 15 % of the total expenses for “thank-you envelops”, thus “portion of expenditure” for “extra thank-you OOP” >15 % is considered to be a high portion of an OOP payment.

**Fig. 3:**
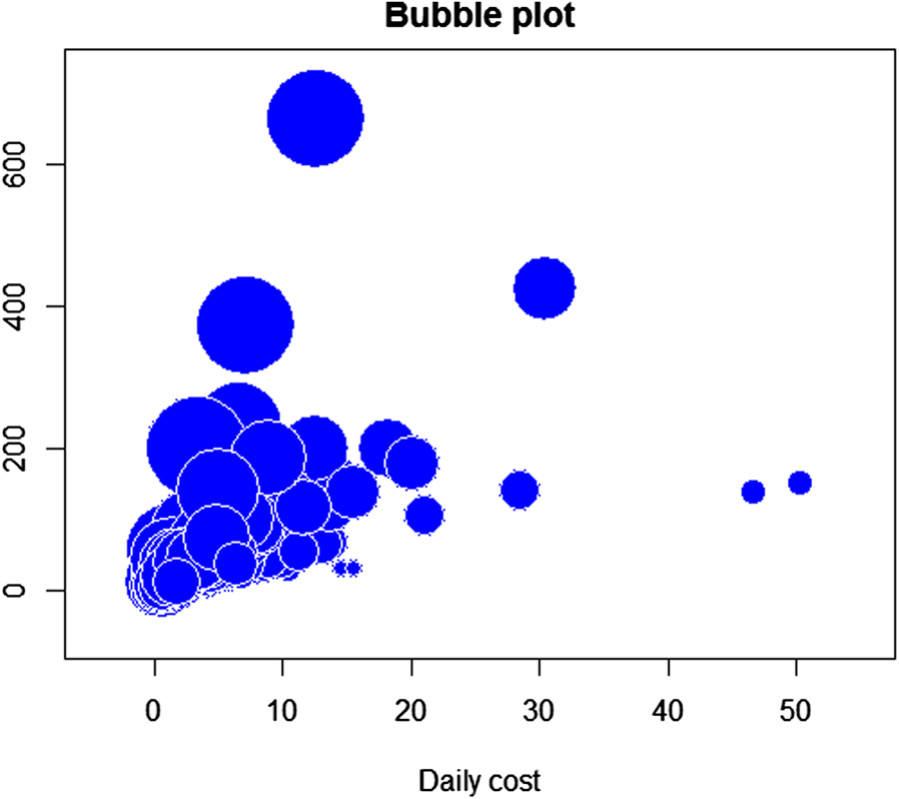
Daily cost, total expenses and days in hospital

Figure 3 represents data points, each with 3 numerical values of average daily cost (horizontal axis; in millions of Vietnamese Dong per day; VND 1 million ~$47.2), total health care expenses for the treatment (vertical axis; in millions of Vietnamese Dong) and number of days in the hospital (taking the natural logarithm to reduce the difference in effect size for better visualisation). The differences among patients are quite substantial.

**Fig. 4:**
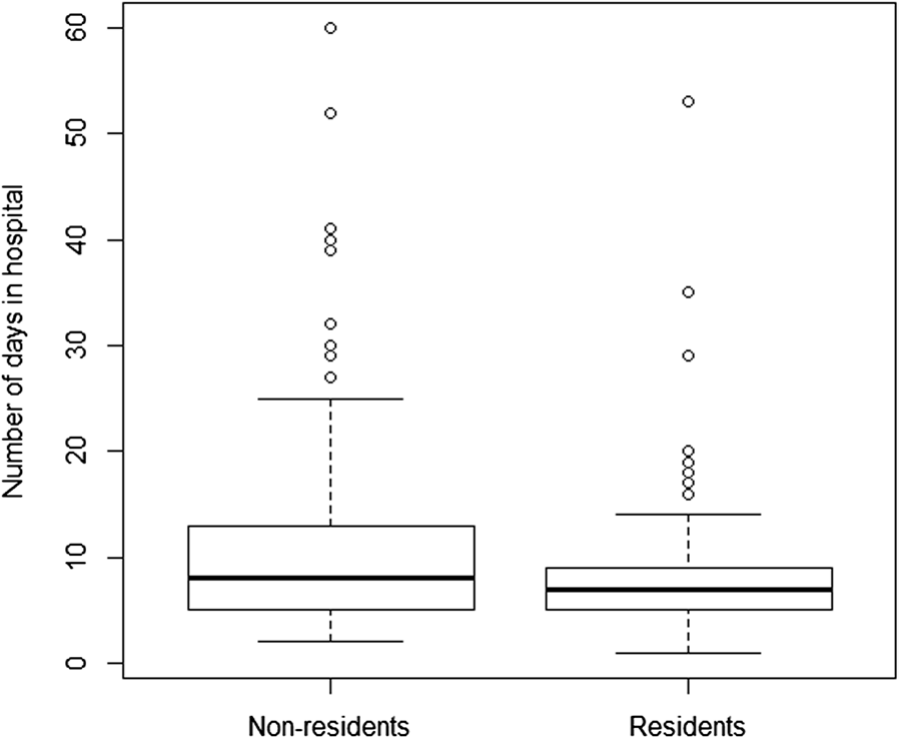
Distribution of days in hospital among patients, subject to status of residency

In Fig. 4, those who were most likely to require longer hospital stays naturally divided into two groups, residents and non-residents. Generally speaking, people coming from other provinces tended to stay a little longer than those from within the region. However, the difference is not very large and likely insignificant. For each group, the dispersion of length of stay was large. In the subsequent analysis, more than a 10-day stay is considered “longer”.

**Fig. 5:**
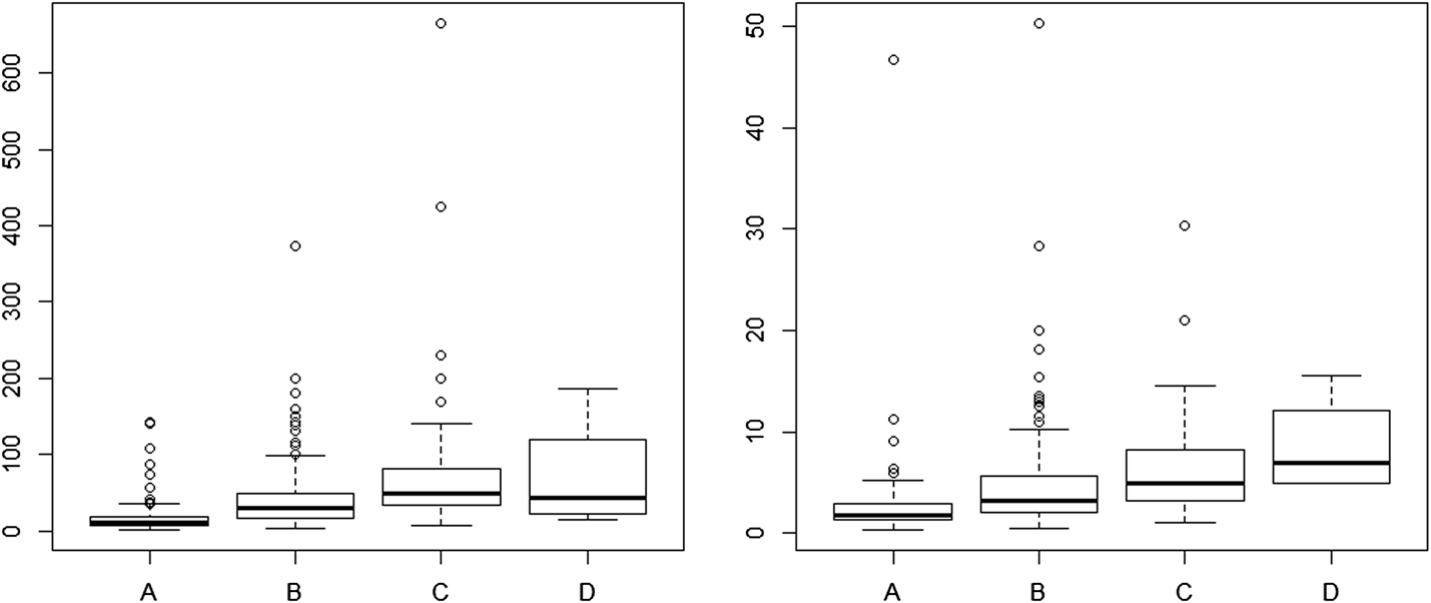
Treatment outcome in relation to expenditure and average daily cost

Next, Fig. 5 provides two graphs for the distribution of total expenses and average daily costs per patient, divided into groups of patients with different end results of treatments (A: full recovery; B: partial recovery; C: stopped in middle; D: unsuccessful treatment, including mortality). Both total expenses and average daily costs are on the vertical axis, and measured in millions of Vietnamese Dong (VND 1 million = US $47.2 using the official exchange rate as of Oct 15, 2014).

For both factors of expenditure and daily cost, the most varying range belongs to group D. There exist outliers if actual monetary values of expenses and daily cost are used. Thus, the choice of categorical data becomes more appropriate.

Additional graphs are provided in “Appendix 2'' for visual checks on possible relationships among pairs of variables. The data set that is used in subsequent estimations and analyses is provided in sub-tables of Table 10 in the “Appendix 3''.

### Results: estimated coefficients, functional forms and probabilities

In what follows, five estimations of joint effects—that likely exacerbate severe impact on probability of destitution—with significant coefficients are reported in separate attempts. In each attempt, coefficients are tabulated, followed by equation forms for stylised facts. Estimated probabilities are computed for the event conditional upon some events specified by the related factors (predictors).

It is noteworthy that in each estimation, no more than two groups of independent categories are used, leading to a limited number of variables entering into specifications. This choice is due to technical requirements for minimum of count value for each cell and the number of cells with count value of less than 5. In addition, as the survey aims at seeking the effect of changes in individual variables rather than comparing them in more complex specifications, a parsimonious specification is preferred in anticipation for better predictive power for computed probabilities.

#### Joint effects of “Residency” and “Insured” on patients’ post-treatment financial distress

This section starts with the first specification, simple but useful for a general public perception, using sub-table BURDEN1 (from Table 10 in "Appendix 3"). Results are provided in Table 1, with all coefficients being statistically significant, mostly at a conventional level (*p* < 0.001).

**Table 1:** Estimation results for probability of distress on “residency” and “insured”

	Intercept	Resident	Insured
		No	No
	β_0_	β_1_	β_2_
Logit (C|A)	−1.1239*** [0.2738] (−4.1046)	2.2628*** [0.3178] (7.1209)	0.9652*** [0.3153] (3.0612)
Logit (B|A)	−0.7349*** [0.2516] (−2.9213)	0.5222* [0.3264] (1.5999)	1.0777*** [0.3349] (3.2181)

Residual deviance: 1.45 on 2 degrees of freedom (df); Log-likelihood: −17.92 on 2 df, Baseline = no financial burden at all; (SE) and z values in parentheses [ ] and ( )

***^,^**^,^* Denote coefficients significant at 1, 5 and 10 %, respectively

Rewriting the above empirical results into the following stylized facts, the first two logits are as follows:

$$ { \ln }\left( {\frac{{\hat{\pi }_{C} }}{{\hat{\pi }_{A} }}} \right) = - 1.1239 + 2.2628NonRes + 0.9652Uninsured $$

$$ { \ln }\left( {\frac{{\hat{\pi }_{B} }}{{\hat{\pi }_{A} }}} \right) = - 0.7349 + 0.5222NonRes + 1.0777Uninsured $$

These logits enable us to estimate the probability that a patient falls into debt if that patient is non-resident and uninsured (or medical costs are not eligible for reimbursement under the policy) $\hat{\pi }_{C}$:

$$ \hat{\pi }_{C} = \frac{{e^{ - 1.1239 + 2.2628 + 0.9652} }}{{1 + e^{ - 1.1239 + 2.2628 + 0.9652} + e^{ - 0.7349 + 0.5222 + 1.0777} }} = 0.7084. $$

The probability that a patient falls into some kind of adverse effect (but not indebtedness) and has negligible or no insurance $\hat{\pi }_{B}$ and is a non-resident:

$$ \hat{\pi }_{B} = \frac{{e^{ - 0.9283 + 1.2128 + 0.7927} }}{{1 + e^{ - 0.9462 + 2.5694 + 0.7642} + e^{ - 0.9283 + 1.2128 + 0.7927} }} = 0.2052. $$

Consequently, only 8.64 % (= 1 − 0.7084 − 0.2052) of non-resident patients will not be adversely affected if hospitalised without insurance. A table for distributions of probabilities follows (Table 2).

**Table 2:** Probability distributions financial burden outcome upon status of residency and eligibility for receiving insurance benefits

Residency	Eligibility	A	B	C
Resident	Yes	0.5542	0.2657	0.1801
	No	0.3065	0.4319	0.2616
Non-resident	Yes	0.2028	0.1639	0.6333
	No	0.0864	0.2052	0.7084

**Fig. 6:**
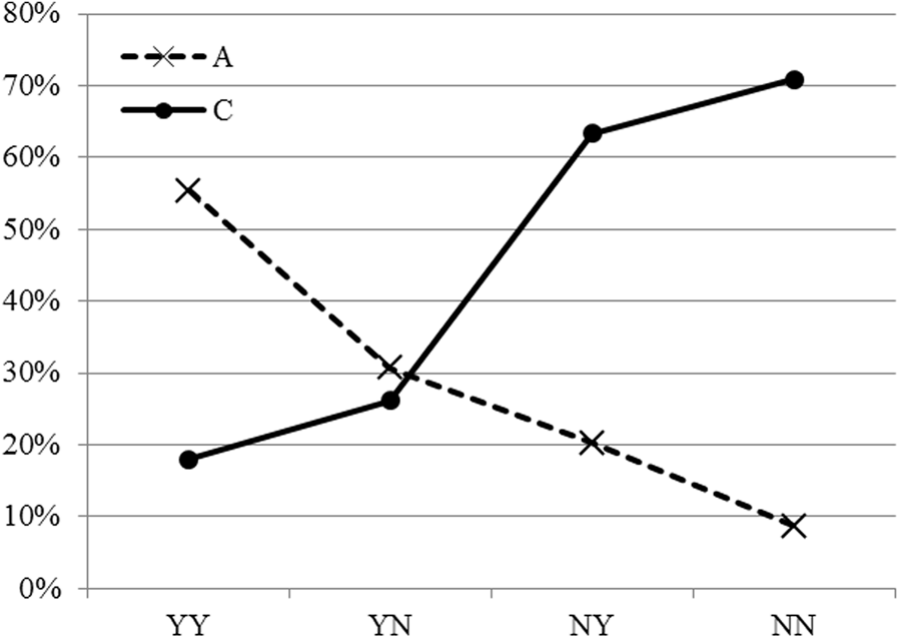
Changing probabilities of burden outcome following status of “Residency” and “Insurance”. *YY* resident and insured; *YN* resident and uninsured; *NY* non-resident and insured; *NN* non-resident and uninsured

From probabilities provided in Table 2, it is straightforward to show the contrast of changing probabilities for different burden outcomes depending on status of residency and eligibility for insurance benefits, illustrated in Fig. 6.

The two lines move in opposite directions. The probability of falling destitute increases when a patient becomes uninsured, but jumps when the patient is non-resident. The highest probability occurs under the joint effect of “uninsured” and “nonresident”. On the other hand, the probability of being minimally affected goes down from the best (>55 %) when a patient is both “insured” and “resident” to the worst (<10 %) when he/she is “uninsured” and “non-resident”.

#### Joint effects of “Insurance benefits” and “Residency” on the probability of distress

The next estimation models the probability of falling into a specific category of post-treatment “financial position”, conditional upon levels of insurance reimbursement and the residency status of the patient and is based on the dataset BURDEN3. Results are provided in Table 3.

**Table 3:** Modeling probability of financial distress upon “residency” and “insurance benefits”

	Intercept	Resident	InsL2		
		No	Lo	Med	Nil
	β_0_	β_1_	β_2_	β_3_	β_4_
Logit (C|A)	−0.9279*** (−2.9276)	2.3381*** (7.2249)	−0.52045 (−1.0214)	−0.6714 (−1.4535)	0.7144** (1.9899)
Logit (B|A)	−0.6466** (−2.0658)	0.5808* (1.7572)	−0.0468 (−0.0858)	−0.5458 (−1.0113)	0.9368** (2.4390)

Baseline = no financial burden at all; z values in parentheses; Residual deviance: 17.31636 on 6 df; Log-likelihood: −35.4262 with 6 df

***^,^ **^,^ * Denote coefficients significant at 1, 5 and 10 % respectively

The above models the probability of falling into burden category C (and B) versus category A (zero adverse effect of health expenditure), depending on whether a patient is a “non-resident” and “uninsured”. The results show a very clear trend. Both burdens of categories C (distressed) and B (partly adversely affected) show significantly negative effects of being a non-resident and having no insurance on patients’ probability of becoming indebted. In other words, having no insurance and being a non-resident increases the log-odds of falling into burden type C or B.

To measure the risk, using results from Table 3, we can take category C (probability of falling into debt) as an example. The non-residency factor has a much larger (negative) effect on the probability of a patient becoming indebted than does being uninsured, with the significant coefficient being +2.388, compared to +0.7144 for being uninsured.

$$ { \ln }\left( {\frac{{\hat{\pi }_{C} }}{{\hat{\pi }_{A} }}} \right) = - 0.9279 + 2.3881NonRes - 0.5204InsLow - 0.6714InsMed + 0.7144InsNil $$

$$ { \ln }\left( {\frac{{\hat{\pi }_{B} }}{{\hat{\pi }_{A} }}} \right) = - 0.6466 + 0.5808NonRes - 0.0468InsLow - 0.5458InsMed + 0.9368InsNil $$

It is then possible to compute the probability that a nonresident patient falling into debt having no insurance $\hat{\pi }_{C}$:

$$ \hat{\pi }_{C} = \frac{{e^{ - 0.9279 + 2.3881 + 0.7144} }}{{1 + e^{ - 0.9279 + 2.3881 + 0.7144} + e^{ - 0.6466 + 0.5808 + 0.9368} }} = 0.6945. $$

The probability of a patient without insurance coming from another region and becoming indebted is quite high, almost 70 %. In addition, the probability that a nonresident patient falling into some kind of adverse effect—but not indebtedness—having no insurance ($\hat{\pi }_{C}$) is much lower, roughly 25 %:

$$ \hat{\pi }_{B} = \frac{{e^{ - 0.6466 + 0.5808 + 0.9368} }}{{1 + e^{ - 0.9279 + 2.3881 + 0.7144} + e^{ - 0.6466 + 0.5808 + 0.9368} }} = 0.2534. $$

Only 5.2 % of non-resident patients will be minimally affected if hospitalised without significant insurance benefits, that is <20 % of total healthcare costs.

#### The effects of “Health Cost” and “Insurance”

Next, consider the probabilities of falling into different financial positions (A = Strong, B = Adversely Affected, or C = Indebted/Destitute) conditional upon levels of average cost of treatment and insurance reimbursements (sub-table BURDEN4). The baseline category for this regression probability has no negative financial effect; the two reference categories for AvgCost and Insurance Level are LowCost and High reimbursement, respectively.

From Table 4, with the exception of the categorical variable, “Medium Insurance” coverage, being insignificant, most coefficients are statistically significant. Stylised facts from Table 4 are rewritten as:

$$ { \ln }\left( {\frac{{\hat{\pi }_{C} }}{{\hat{\pi }_{A} }}} \right) = - 0.9462 + 2.5694HiCost + 1.2158MedCost - 0.1436InsMed + 0.7642InsNeg $$

$$ { \ln }\left( {\frac{{\hat{\pi }_{B} }}{{\hat{\pi }_{A} }}} \right) = - 0.9283 + 1.2128HiCost + 0.5020MedCost - 0.3656InsMed + 0.7927InsNeg $$

These can be converted into the probability of a patient falling into debt and having negligible insurance benefits while paying high health care cost ($\hat{\pi }_{C}$) is as follows:

$$ \hat{\pi }_{C} = \frac{{e^{ - 0.9462 + 2.5694 + 0.7642} }}{{1 + e^{ - 0.9462 + 2.5694 + 0.7642} + e^{ - 0.9283 + 1.2128 + 0.7927} }} = 0.6912. $$

The probability that a patient falling into some kind of adverse effect (but not indebtedness) and having negligible or no insurance $\hat{\pi }_{B}$, while paying higher cost of services is:

$$ \hat{\pi }_{B} = \frac{{e^{ - 0.9283 + 1.2128 + 0.7927} }}{{1 + e^{ - 0.9462 + 2.5694 + 0.7642} + e^{ - 0.9283 + 1.2128 + 0.7927} }} = 0.2645. $$

Only 4.43 % of patients will not be adversely affected if hospitalised without insurance while paying higher costs. From the results of Tables 3 and 4, it is safe to state that the joint effect of “non-residency” + “uninsured” has a similar impact on risk of destitution (and hardship) as the joint effect of “receiving negligible benefits” + “high costs of health services”. To obtain a more interesting finding to see if low insurance benefits are as much of a risk as being uninsured when costs are high, an additional estimation is performed and results are provided in “Appendix 4''. Since only β_5_ is significant, it is not decisive to compare the two cases.

**Table 4:** Modeling categories of financial burden following average cost and insurance levels

	β_0_	AvgCost		Insurance level	
		HiCost	MedCost	Med	Neg
		β_1_	β_2_	β_3_	β_4_
Logit (C|A)	−0.9462** (−2.5037)	2.5694*** (5.1764)	1.2158*** (3.3389)	−0.1436 (−0.3233)	0.7642** (2.3656)
Logit (B|A)	−0.9283** (−2.3424)	1.2128** (2.2110)	0.5020 (1.3182)	−0.3656 (−0.6778)	0.7927** (2.1660)

Residual deviance: 5.72 on 8 df; Log-likelihood: −32.19 on 8 df z value in brackets. Baseline category: no financial burden after staying in hospital

***^,^**^,^* Denote coefficients significant at 1, 5 and 10 %, respectively

Next, Table 5 presents the probabilities of falling into different financial positions conditional upon status of insurance (“Insured” and “Uninsured”) and Average Cost (“High” and “Medium”, and “Low”) (sub-table INSURANCE “Appendix 3'').

**Table 5:** Modelling categories of financial burden following average cost and insurance status

	β_0_	AvgCost		Insurance
		High cost	Med. cost	Uninsured
		β_1_	β_2_	β_3_
Logit (C|A)	−1.0346** [−3.070]	2.6368*** [5.279]	1.2653*** [3.459]	1.1261*** [3.689]
Logit (B|A)	−1.0328** [−3.018]	1.2865* [2.342]	0.5621 [1.477]	1.1223*** [3.316]

Residual deviance: 10.91 on 4 df; Log-likelihood: −28.18 on 4 df z value in brackets. Baseline category: no financial burden after staying in hospital

***^,^ **^,^ * Denote coefficients significant at 1, 5 and 10 %, respectively

Most coefficients in Table 5, with the exception of “MedCost,” are statistically significant. In light of this, financial burdens after treating in hospital are rewritten as:

$$ { \ln }\left( {\frac{{\hat{\pi }_{C} }}{{\hat{\pi }_{A} }}} \right) = - 1.0346 + 2.6368HiCost + 1.2653MedCost + 1.1261Uninsured $$

$$ { \ln }\left( {\frac{{\hat{\pi }_{B} }}{{\hat{\pi }_{A} }}} \right) = - 1.0328 + 1.2865HiCost + 0.5621MedCost + 1.1223Uninsured $$

The conditional probability of patients who are falling into indebtedness (category C) while paying high costs and having no insurance is as follows:

$$ \hat{\pi }_{C} = \frac{{e^{ - 1.0346 + 2.6368 + 1.1261} }}{{1 + e^{ - 1.0346 + 2.6368 + 1.1261} + e^{ - 1.0328 + 1.2865 + 1.1223} }} = 0.7553. $$

The above results suggest that when uninsured patients have to pay higher costs their probability of going destitute becomes ~75.5 %, significantly higher than the probability of 69.1 % as derived from Table 4. Furthermore, the joint effects of paying high cost and being (in) eligible for insurance benefits on probabilities of different burden outcomes, computed from Table 5, are shown by the distributions in Table 6.

**Table 6:** Summary of probabilities of destitution on “Insurance” and “High Cost”

Burden	A	B	C	A	B	C
Avg. cost	Uninsured			Insured		
High	0.0493	0.1954	0.7553	0.1379	0.1777	0.6844
Low	0.3135	0.3429	0.3436	0.5843	0.2080	0.2077
Medium	0.1470	0.2821	0.5709	0.3467	0.2166	0.4367

**Fig. 7:**
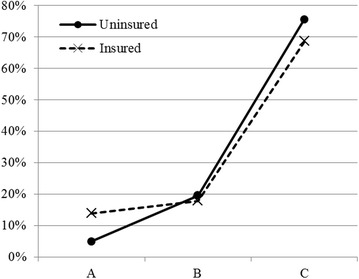
Changing probabilities of category A and C burden outcome following high costs and eligibility of insurance

To visualize the effects of changes in level of health costs and eligibility for receiving insurance benefits, Fig. 7 may be useful. Regarding best outcome (A), when facing high costs of treatment, the probability for the “uninsured” falls by almost 9 percentage point from that of the “insured” (13.8–4.9). But for the worst outcome (C), the gap is narrower: (75.5–68.4) = 7 percentage point. Comparatively, the effect of change in A, when a patient moves from “insured” to “uninsured”, is much stronger (9/13.8) than in C (7/75.5). That is to say the negative effect of being uninsured is rather ‘stable’ in determining the probabilities of going destitute.

The slopes of two lines in Fig. 7 also indicate that the “uninsured” is generally disadvantageous compared to the insured, with respect to the distinct outcomes A and C that the survey wants to observe. For the moderately affected cases (category B), the difference appears to be quite negiligible.

#### Treatment outcome, health care cost and severity of illness

The next analysis focuses on the probabilities of “Treatment Outcome” for patients, conditional upon average cost of services and severity of illness, employing the END2 subset from Table 10 in the "Appendix 3". Estimated coefficients are provided in Table 7, following which, most coefficients are highly significant. Table 7 results are rewritten in equation forms as follows:

$$ { \ln }\left( {\frac{{\hat{\pi }_{C} }}{{\hat{\pi }_{A} }}} \right) = - 4.5965 + 2.3444Bad + 4.1486Emergency + 3.2568HighCost + 1.2881MedCost $$

$$ { \ln }\left( {\frac{{\hat{\pi }_{B} }}{{\hat{\pi }_{A} }}} \right) = - 1.1231 + 1.1452Bad + 1.1074Emergency + 2.4116HighCost + 1.08MedCost $$

The results show that the probability that a patient quits while in a condition of “serious illness” and anticipating expensive treatments ($\hat{\pi }_{C}$) is quite high, 58 %:

$$ \hat{\pi }_{C} = \frac{{e^{ - 4.5965 + 4.1486 + 3.2568} }}{{1 + e^{ - 4.5965 + 4.1486 + 3.2568} + e^{ - 1.1231 + 1.1074 + 2.4116} }} = 0.5805. $$

Likewise, the probability that a patient can only be partially cured $\hat{\pi }_{B}$ while paying high cost of services is also substantial, over 38 %:

$$ \hat{\pi }_{B} = \frac{{e^{ - 1.1231 + 1.1074 + 2.4116} }}{{1 + e^{ - 4.5965 + 4.1486 + 3.2568} + e^{ - 1.1231 + 1.1074 + 2.4116} }} = 0.3845. $$

In the estimated $\hat{\pi }_{C}$, both conditions of illness and high costs have a large impact on increasing the risk of early quitting. However, in case of $\hat{\pi }_{B}$ costliness of treatment appears to be the more determining factor.

**Table 7:** Modeling “Treatment Outcome” following cost levels and illness

	Intercept	Illness		Average cost of services	
		Bad	Emergency	High	Medium
	β_0_	β_1_	β_2_	β_3_	β_4_
Logit (C|A)	−4.5965*** (−4.9401)	2.3444*** (2.9511)	4.1486*** (5.0950)	3.2568*** (4.0096)	1.2881* (1.8532)
Logit (B|A)	−1.1231*** (−3.5675)	1.1452*** (3.8388)	1.1074*** (2.6608)	2.4116*** (4.6114)	1.0800*** (3.2739)

Residual deviance: 14.36 on 8 df; Log-likelihood: −32.17 on 8 df z value in brackets. Baseline category: complete recovery after treatments

***^,^**^,^* Denote coefficients significant at 1, 5 and 10 %, respectively

These two high probabilities lead to the fact that the probability of full recovery for a patient hospitalised with an emergency, anticipating higher costs of treatment, is very low, just 3.5 %.

#### On the sensitive issue of “extra thank-you money” OOP

Finally, estimation results are reported based on data provided in the ENV2 sub-table, which model the probability of a patient paying high or medium “extra thank-you money” conditional upon income ranks and/or severity of illness. The baseline category is “paying negligible thank-you” for the response variable. For “Ill2” the reference category is “light sickness” and for “Income Rank”, the reference is “Medium”.

Clearly, the estimated results in Table 8 show that both income ranks of the category Low (of “Income Rank”) and both Bad and Emergency (of “Illness”) jointly reduce the probability of patients paying “thank-you money” from medium to high level. That is, these lower-income patients, when faced with serious illness or emergency, are less likely to be able to afford significant “Extra thank-you money” OOP payments.

$$ { \ln }\left( {\frac{{\hat{\pi }_{HiPay} }}{{\hat{\pi }_{NegPay} }}} \right) = + 0.5079 - 1.7684Bad - 1.4804Emerg - 0.5079HiInc - 0.9134LowInc $$

$$ { \ln }\left( {\frac{{\hat{\pi }_{MedPay} }}{{\hat{\pi }_{NegPay} }}} \right) = + 0.6104 - 1.0751Bad - 1.4605Emerg - 0.9899HiInc - 1.0804LowInc $$

Still, the poor who have an “emergency” are willing to make an expensive “thank-you” OOP payment with a probability ($\hat{\pi }_{HiPay}$) of 7.8 % following the above estimation:

$$ \hat{\pi }_{HiPay} = \frac{{e^{ - 1.4804 - 0.9134} }}{{1 + e^{ - 1.4804 - 0.9134} + e^{ - 1.4605 - 1.0804} }} = 0.0780. $$

Likewise, $\hat{\pi }_{MedPay}$ is estimated at ~6.7 %:

$$ \hat{\pi }_{MedPay} = \frac{{e^{ - 1.4605 - 1.0804} }}{{1 + e^{ - 1.4804 - 0.9134} + e^{ - 1.4605 - 1.0804} }} = 0.0673. $$

After all, it is with a high probability, 85.46 %, that low-income patients with an emergency can only afford a negligible amount of “extra thank-you” OOP payment.

**Table 8:** Modeling “Extra thank-you money” against “Illness” and “Income Rank”

	β_0_	Illness		Income rank	
		Bad	Emergency	High	Low
		β_1_	β_2_	β_3_	β_4_
Logit (HiPay|Neg)	0.5079 [0.5813] (0.8737)	−1.7684*** [0.4560] (−3.8778)	−1.4804*** [0.4851] (−3.0520)	−0.5079 [0.6657] (−0.7629)	−0.9134* [0.4835] (−1.8891)
Logit (MedPay|Neg)	0.6104 [0.5128] (1.1902)	−1.0751*** 0.41346 (−2.6003)	−1.4605*** [0.4880] (−2.9930)	−0.9899 [0.6265] (−1.5799)	−1.0804*** [0.3933] (−2.7471)

Residual deviance: 3.81 on 2 df; Log-likelihood: −24.11 on 2 df

[SE] in square bracket and (z value) in parentheses. Baseline category: pay negligible “extra envelop” amount

***^, ^**^, ^* Denote coefficients significant at 1, 5 and 10 %, respectively

## Discussion and concluding remarks

The attempts to model the risk of destitution among patients suggest that that key variables are crucial. The following discusses those relationships, concluding remarks, and implications for policy making and the efficient use of insurance by patients.

Clearly, being hospitalized without insurance represents a high risk of going destitute for all in-patients. The chance of being minimally affected in terms of financial situation after expensive in-patient improves significantly for insured patients. The practice of travelling from rural areas to large cities, in hopes of better healthcare services also adds to the probability of becoming destitute. This is because of unexpected costs such as informational asymmetry, and travel costs. It is highly probable that for every three uninsured and non-resident hospitalised patients, two will face serious financial hardship or destitution. Further, results suggest that for every two patients hospitalised with a serious illness requiring costly treatment, it is highly likely that at least one would risk going destitute. Although researchers would say the risk is high, an important question is “how high is high” to provide the public and policy-makers with more insightful answers. These probabilities suggest that Vietnamese patients are more vulnerable to the risk of destitution than previously thought.

The finding that low-income patients with emergency or serious illness are less likely to give high amounts of “extra thank-you money” suggests that the amount is a small proportion of treatment cost. “Thank-you money” may be a cultural norm showing patients’ respect for people taking care of their health or even saving their lives. In light of this, physicians should accept this respectable gesture. Moreover, as the probability of full recovery is low, patients—especially those are hospitalized with an emergency—are more willing to make good relationships with hospital staff members since they are concerned about a future hospital visit.

Other forms of OOP expenses may include “payment” for access to government-funded programs, such as treatments with advanced equipment and/or expensive drugs. Selvaraj (2010) reveals that OOP spending has increased in India due to a substantial surge of drug prices over the years. Such practices may make the programs—i.e., health care insurance—end up with a narrower focus than original mandated, even widening the gaps between groups of patients in different levels of income. In addition to coverage expansion, further health care reforms should improve the equality of distribution of benefits (Chen et al. 2014, 2015). Further, it could alleviate the moral hazards of beneficiaries with high health insurance coverage levels (Kim et al. 2015). Understanding these challenges calls for further research.

Given the fact that only 5.2 % of non-resident patients will be minimally affected if they are hospitalised without significant insurance benefits, popular bypassing may exacerbate rural–urban disparities. Indeed, it has happened in Malaysia (Loganathan et al. 2015) and China (Hu et al. 2008), where most medical resources are allocated to urban areas. In such a circumstance, inequalities are hardly narrowed by expansions of health insurance coverage and service utilization (Fu et al. 2014).

In addition to travelling and accommodation burdens, bypassing patients face asymmetric information. The patients, particularly who are poor and come from rural areas, often make OOP payments to access allegedly better medical treatment even if they risk falling into severe indebtedness. Their “bargaining power” is weak as they usually make payment without asking for “deliverables”. Limited information, which is collected from small group of patients whose situations are similar, may lead the uninsured to overpay but also to not have access to medicines being abundant in Vietnam (Mao et al. 2015). The patients may make OOP payments for special and advanced medicines without knowing whether those medicines are needed or likely to help. Their judgments are almost based on price and scarcity of the medicines so they believe the more expensive and rare the medicines are, the more effective they must be. Such misleading perception may add unnecessary, but possibly expensive, expenses to the treatment costs. The expenses, in turn, raise the probability of being destitute.

For health services providers, including hospitals, the results suggest that their future depends on the payment servicing capacity, and the risk is high for patients when costs are high. The risk is exacerbated if for some reasons (in fact, the reasons are countless) a patient is not eligible for a substantial portion of insurance package that he/she is entitled to, in principle, ~70 %. Leaving patients destitute after treatments, due to high costs and inadequately covered by insurance, could damage the hospital’s reputation and future. This implies a more proactive coordination between hospitals and health insurance authorities in alleviating the obstacles to eligible rights of the patients.

As the amended Law on Health Insurance comes into effect, the ambitious plan of aiming at 100 % UC and all Vietnamese having health insurance faces a dilemma. While the current statistics show that roughly 60 % of Vietnamese hold UC, the majority of insured patients could hardly be financed adequately by insurance, see Fig. 2a, b. One could only guess what would happen if this current level of 60 % insured increased to 100 %; it is likely that the actual coverage range would go down. If this decrease would lead to higher rate of “negligible insurance”, the computed probabilities would enable us to predict that the probability of falling into destitution may even rise. In fact, before the introduction of the new law, tension already ran high at times in the 2013–2014 period upon news of a possible collapse of the Vietnam Health Insurance Fund, causing deep concerns in society. This says that, without an appropriate evidence-based policy making process, an idea that is nice initially may eventually end up penalising the poor.

## Statement of research ethics:

I ensure the basic principles of ethical research as stated below:

- Beneficence: As a researcher I strive to ensure that my work makes a positive contribution to the welfare of those affected by it.
- Non-malfeasance: I endeavour to ensure that the research work does not cause harm to any sectors of society and, in particular, to participants.
- Justice: The benefits and risks associated with this study should be well assessed in advance and both should be equitably distributed throughout society.
- Autonomy of subjects: The research respects and protects the rights and dignity of participants.

As the research involves primary data collection, in the form of interview surveys, I have been fully compliant with all legal requirements regarding the collection, storage, handling, processing and analysis of data. I guarantee that I have conformed to the highest standards of:

- Veracity: Participants in sample surveys and data collection exercises are given full and accurate information regarding issues such as the background, nature, purpose, and outputs of the research.
- Informed consent: Participants in sample surveys and related data collection exercises were given sufficient details on the research in question as to allow them to make an informed decision to participate or otherwise in a research study.
- Protection of vulnerable groups: I am particularly conscious of my obligations to safeguard the interests of vulnerable or potentially ‘at risk’ groups who may be involved in my research.
- Privacy: Participants in data collection exercises have the right not only to agree to participate in the research but also to decide to withdraw from the research at any time.
- Confidentiality: The information provided by participants is treated as confidential and used for research purposes only. Micro-level information will not be disclosed in any fashion to third parties, which would allow it to be associated with an identifiable individual.
- Minimising risk: Participants in the research will not be put under undue or unnecessary risk as a result of their participation.
- Research outputs: I am committed to putting the results of this research into the public domain (always on an anonymous basis) with a view to transparency, scrutiny and peer review. Where feasible, I am committed to depositing anonymous primary data collected in the course of research into a publicly accessible data archive.

## Figures and Tables

**Fig. 8:**
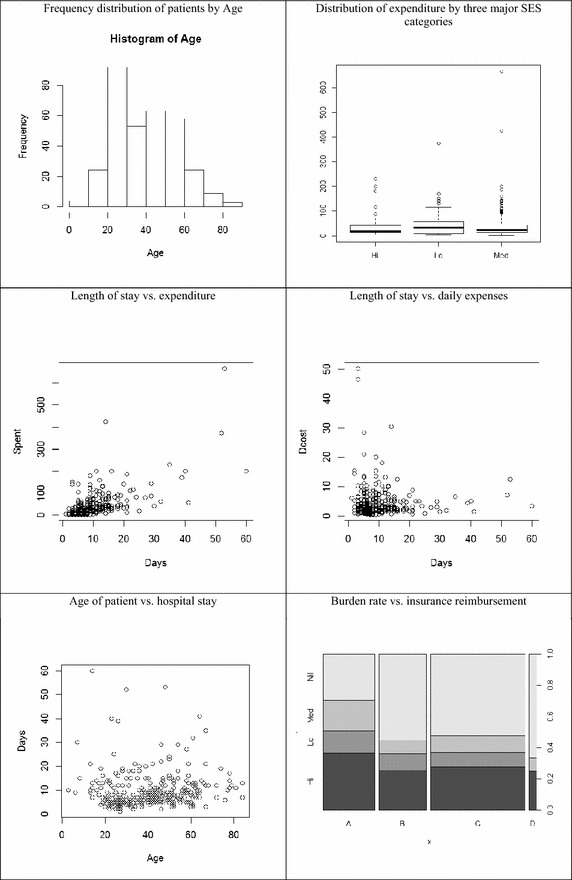
Supplementary graphs for visualisation of data set

**Fig. 9:**
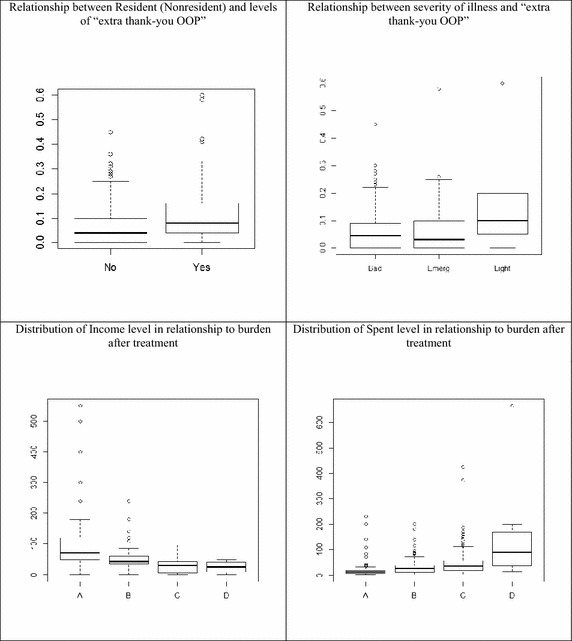
Supplementary graphs for visualisation of data set

## Appendix 1

See Table 9.

**Table 9:** Reading data set into the R system

Factor	Basic description	Characteristics	Remark for later use
ID	Coded number for each patient	Each patient has a unique code	This will only be used to produced graphs where needed
Name	Name of patient	Those who refuse to allow the use of their true name will be replaced by N.A. Some patients may have identical names, and they will be distinguished by ID	This will not be used
Res	This answers if the patient is resident of the region where the hospital is located	This takes binary value of yes or no	This has a potential value for implying transports and related costs for patient’s relatives
Days	Number of days the patient spent in the hospital	Quantitative variable	This is for charting the frequency distribution and transforming into long or short stay
Stay	A dummy variable to define if length of stay in hospital by a patient is short or longer	This takes value of S if one’s stay is less than 10 days, and L (longer) if 10 days or longer	This is potentially a good candidate for a binary predictor variable
Insured	Whether a patient has a valid health insurance policy	This variable is binary and takes value of “Yes” or “No”	This is potentially a good candidate for a binary predictor variable
MaxIns	The maximum level of coverage by insurance policy if the patient has one	Quantitative factor, measured by percentage of total “eligible costs” according to regulations	This is supplementary information only
Edu	Highest level of education	Values: JHS: junior high HS: high school Uni: college/university Grad: graduate school	This is supplementary information only
SES	Socio-economic status	This is a multicategory variable, taking values of: high, medium, low	This is potentially a good candidate for a multi-categorical predictor variable
Illness	Degree of severity of illness or injury when hospitalized	This is a multicategory variable, taking values of: emergency, bad, ill and light	This is potentially a good candidate for a multi-categorical predictor variable. Ill and Light categories can also be grouped into one single category, in which case we have a transformed factor of Ill2 (also in the data set)
Jcond	Status of job	This takes value of good, stable, unstable, unemployed or N.A. (i.e. others: retired, high school students)	This is supplementary information only
Income	Annual income in millions of Vietnamese Dong. Current exchange rate: $1 = VND21,200 (as of Dec 1, 2014)	Quantitative factor. The factor can be continuous in theory, but is mostly categorical in practice	This will be used to derive the IncRank variable
IncRank	Rankings of income of a patient	This takes value of high, middle, or low	This is potentially a good candidate for a multi-categorical predictor variable
Spent	Total money spent during his/her stay in hospital in millions of Vietnamese Dong. Using official exchange rate, VND 1 million is equivalent to $47.2	Quantitative variable	
Dcost	Average daily cost the patient had to pay during treatment period	Quantitative variable	
Pins; Pinc; Pchar; Ploan	Portions of finance from sources: insurance reimbursement, income, charity funds from civil organizations or employers, or borrowings	Quantitative variables. They are measure in percentage of total costs the patient had to cover	
InsL2	Ranked levels of actual insurance reimbursement for patients who have policies	It takes value of high, medium, or low if a patient has an insurance policy. It takes nil if the patient is not insured at all	For actual transformation when necessary both low and nil can be grouped into one category of Neg (i.e., negligible)
Streat, Srel, Senv	Percentage of funds used for the purpose of main treatments, for covering costs of relatives coming to help the patient, or paying “extra thank-you money” or bribing doctors/staff	These are quantitative variables, taking value of percentage. For instance, patient ID001 has {89;4;7 %} = {0.89; 0.04; 0.07}. Streat + Srel + Senv = 100 %	Only ‘Senv’ can be a potential candidate for modeling as dependent categorical variable, after being transformed into levels of “extra fees”, taking value of high, medium, or low. that newly derived categorical variable is called EnvL and also appears in the data set
Burden	Patient’s and family’s self-evaluation of their financial position after paying health care costs	This is a multi-category variable, taking value of A (strong; no adverse affect at all); B (affected but not the worrying level); C (seriously affected) and D (destitute/“bankrupt”	This represents a group of critically important categories that the study employs to learn about what factors likely affect the probabilities of falling into each category of financial burden
End	The health status after treatment	This is a group of multi-categorical variables, taking values of A (complete recovery), B (partial recovery, needing post-treatment follow-ups), C (stopped whilst being treated), or D (quit early)	This represents a group of critically important categories that the study wants to model to know what factors affect the probabilities of falling into each category of treatment completion after patients’ stay in hospital
IfHigher	Self-evaluation of patient and family about financial status if the patient continues to be hospitalised again	This takes the same values as “Burden” factor	This can potentially be a candidate for future examination, especially when a larger sample is available. It is not used for this analysis

## Appendix 2

See Figs. 8 and 9; Additional file 3.

## Appendix 3: The data set and contingency tables

The data set used in this analysis is structured into 5 contingency tables (called “sub-tables”) containing categorical response and predictor variables produced for relevant estimations, and corresponding count values. Each sub-table is given a name for easier reference in upcoming discussions (see Table 10).

**Table 10:** The data set and contingency tables

## BURDEN3 InsL2 Res A B C Hi No 5 9 41 Hi Yes 26 11 5 Med No 11 1 16 Med Yes 6 6 2 Nil No 11 24 72 Nil Yes 14 19 17 Lo No 4 5 13 Lo Yes 8 3 1	## BURDEN4 AvgCost InsL2 A B C HiCost Hi 3 4 22 HiCost Med 1 1 8 HiCost Neg 4 9 24 MedCost Hi 15 13 19 MedCost Med 8 3 8 MedCost Neg 24 32 69 LowCost Hi 13 3 4 LowCost Med 8 3 2 LowCost Neg 9 10 10
## BURDEN1 ## Resident Insured A B C Nonres No 9 23 71 Nonres Yes 22 16 71 Res No 14 18 14 Res Yes 40 21 11	## END2 Ill2 AvgCost A B C Bad Hi 3 33 6 Bad Med 17 59 9 Bad Lo 12 8 1 Emerg Hi 0 8 14 Emerg Med 6 24 17 Emerg Lo 5 1 1 Light Hi 3 8 1 Light Med 34 26 0 Light Lo 21 12 1
## ENV2 Ill2 IncRank Hi Med Nil Bad HM 7 13 17 Bad L 8 17 86 Emerg HM 1 2 9 Emerg L 9 8 47 Light Hi 19 13 19 Light L 16 15 24	##INSURANCE Insured AvgCost A B C No Hi 4 7 17 No Lo 4 9 10 No Med 15 25 58 Yes Hi 4 7 37 Yes Lo 26 7 6 Yes Med 32 23 39
##INSLEVEL InsL2 AvgCost A B C Hi Hi 3 4 22 Hi Lo 13 3 4 Hi Med 15 13 20 Lo Hi 0 2 6 Lo Lo 4 1 0 Lo Med 8 5 8 Med Hi 1 1 8 Med Lo 8 3 2 Med Med 8 3 8 Nil Hi 4 7 18 Nil Lo 5 9 10 Nil Med 16 27 61	

Checked against the data structure and requirement for minimal count value of a cell and all cells, particularly for polytomous logistic regression, the empirical data presented in these sub-tables are satisfactory and ready for estimations

## Appendix 4

See Table 11.

**Table 11:** Test results on relationship between “Average Cost” and “Insurance Levels”: Reported coefficients are estimated using the data of contingency table INSLEVEL (see “Appendix 3'')

Modelling categories of financial burden following Average Cost and Insurance Level	β_0_	AvgCost		Insurance level		
		HiCost	MedCost	Med	Low	Nil
		β_1_	β_2_	β_3_	β_4_	β_5_
Logit (C|A)	−0.9515* (−2.505)	2.631*** (5.255)	1.2503*** (3.397)	−0.1669 (−0.375)	−0.2665 (−0.549)	1.0133** (2.933)
Logit (B|A)	−0.9454* (−2.375)	1.2636* (2.292)	0.5224 (1.361)	−0.3641 (−0.674)	0.0053 (0.010)	1.0263** (2.642)

Residual deviance: 13.64 on 12 df; Log-likelihood: −40.81 on 12 df z value in brackets. Baseline category: no financial burden after staying in hospital

***^,^**^,^* Denote coefficients significant at 1, 5 and 10 %, respectively

## Additional files

**Additional file 1:** Analysis of patients survey 2014 (330 cases).
**Additional file 2:** Data tables for estimations.
**Additional file 3:** Research ethics compliance.
