# Supplementary material for: Be rich or don’t be sick: estimating Vietnamese patients’ risk of falling into destitution
Source: Springerplus. 2015 Sep 21;4:529. doi: 10.1186/s40064-015-1279-x (PMC4577521; doi:10.1186/s40064-015-1279-x)
Supplement: Supplementary file 1 — Additional file 1: Analysis of patients survey 2014 (330 cases). [file 40064_2015_1279_MOESM1_ESM.pdf]

©2014 Q.H. Vuong

Analysis of Patients Survey 2014 (330 cases)

Input data into the system (R)

```
> pdat <- read.table("c:/Dr.Vuong/PS/P330.csv", sep=",",header=TRUE)
> head(pdat)
> head(pdat)
    ID               Name Sex Age Res Days Stay Insured MaxIns  Edu SES Illness
1 P001    Dao Trong Phuc   M  50  No    8    S      No   0.00   HS Med     Bad
2 P002  Nguyen Ngoc Long   M  50  No    6    S      No   0.00  Uni  Hi     Bad
3 P003 Nguyen Hoang Sinh   M  37  No    9    S      No   0.00 Grad  Hi     Bad
4 P004      Dau Thi Que    F  67  No   12    L     Yes   0.95   HS Med     Bad
5 P005     Tran Van Tuan   M  22 Yes    6    S     Yes   0.00  Uni  Lo     Bad
6 P006  Nguyen Manh Tuan   M  18  No   19    L      No   0.00   HS  Lo   Emerg
   Ill2  Jcond WkYrs Income Saving IncRank AvgCost Dcost Spent Pins Pinc Pchar
1   Bad Unstab    20     42    0.1      Lo     Med   3.5    28 0.00 0.90     0
2   Bad   Stab    25    120    0.2     Mid     Med   3.5    21 0.00 1.00     0
3   Bad   Stab     7    180    0.3     Mid     Med   2.1    19 0.00 1.00     0
4   Bad   Stab    35     42    0.0      Lo     Med   4.1    49 0.43 0.40     0
5   Bad   Stab     1     72    0.5     Mid     Med   3.7    22 0.00 0.50     0
6 Emerg  Unemp     0      0    0.0      Lo     Med   2.2    42 0.00 0.95     0
  Ploan InsL InsL2 LoanL Streat Srel Senv EnvL Burden End SatIns SatServ
1  0.10 N.E.   Nil    Lo   0.89 0.04 0.07   Lo      B   B    Avg       B
2  0.00 N.E.   Nil   Nil   0.86 0.05 0.09  Med      A   A    Avg       A
3  0.00 N.E.   Nil   Nil   0.79 0.05 0.16   Hi      A   A    Avg       A
4  0.17    B   Med    Lo   0.93 0.03 0.04   Lo      C   B    Avg       B
5  0.50 N.E.   Nil    Hi   0.91 0.02 0.07   Lo      C   B    Avg       B
6  0.05 N.E.   Nil    Lo   0.88 0.05 0.07   Lo      C   B     Lo       B
  IfHigher InsGap Hospital
1        C   0.00
2        A   0.00
3        A   0.00
4        B  -0.52
5        C   0.00
6        C   0.00
>
```

Descriptive analysis

```
> attach(pdat)
> attach(pdat)
> tab.resinsburden.1 <- xtabs(~Res+Insured+Burden, data=pdat)
> ftable(tab.resinsburden.1)
            Burden  A  B  C  D
Res Insured
No  No              9 23 64  7
    Yes            22 16 67  4
Yes No             14 18 13  1
    Yes            40 21 11  0
>
```

©2014 Q.H. Vuong

Histograms

```
> hist(Days)
```

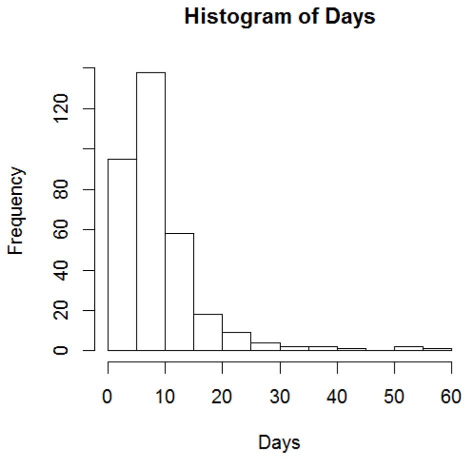


```
> hist(Pins)
```

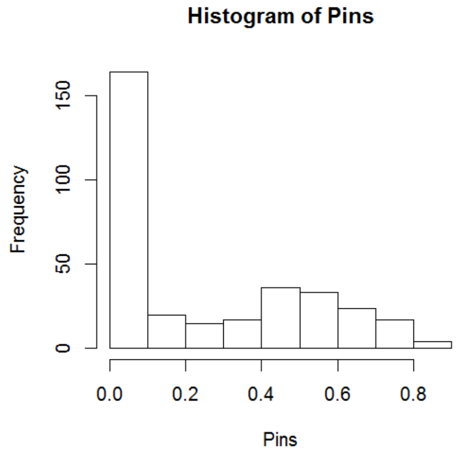


```
> hist(Pinc)
```

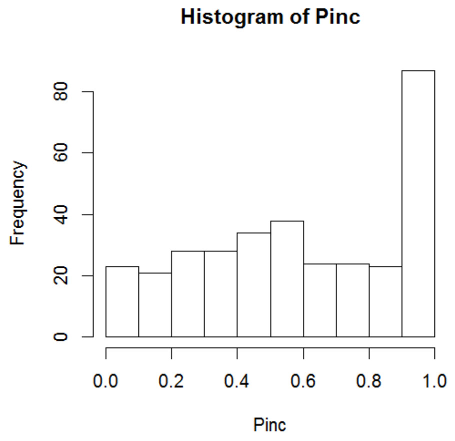


```
> hist(Senv)
```

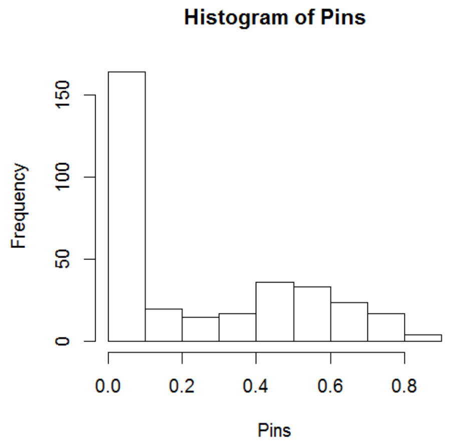


```
> hist(Streat)
```

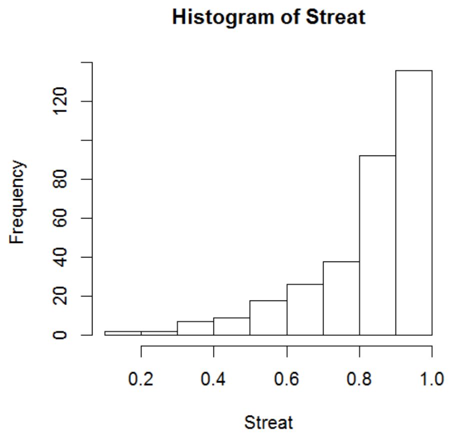


```
> hist(Ploan)
```

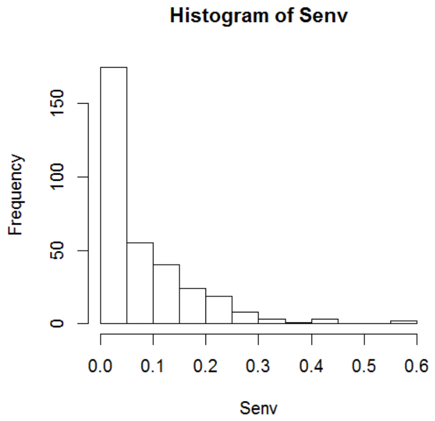

©2014 Q.H. Vuong

```
> hist(Age)
```

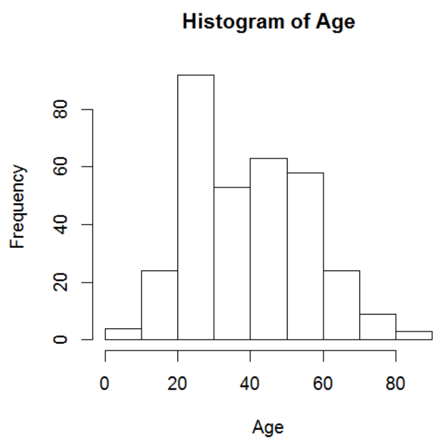


```
> hist(Income)
```

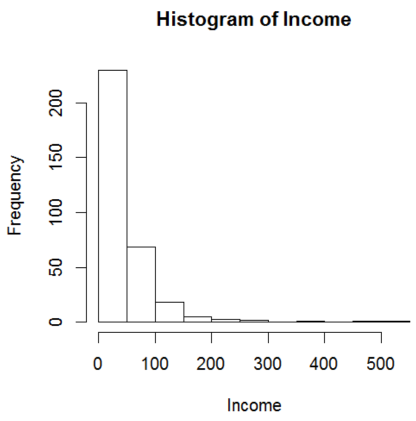

Contingency tables for subsequent modeling (polytomous logistic models)

```
> tab.burden.1 <- xtabs(~InsL2
+IncRank+Burden, data=pdat)
> ftable(tab.burden.1)
             Burden  A  B  C  D
InsL2 IncRank
Hi    Hi             3  0  0  0
      Lo            10 14 38  3
      Mid           18  6  5  0
Lo    Hi             2  0  0  0
      Lo             1  7 13  0
      Mid            9  1  1  0
Med   Hi             1  1  0  0
      Lo             6  5 17  1
      Mid           10  1  0  0
Nil   Hi             1  0  0  0
      Lo             7 26 74  8
      Mid           17 17  7  0
>
```

```
> tab.burden.2 <-
xtabs(~AvgCost+Stay+Burden, data=pdat)
> ftable(tab.burden.2)
             Burden  A  B  C  D
AvgCost Stay
Hi      L            2  4 14  5
        S            6 10 33  2
Lo      L            5  4  7  1
        S           25 12  8  0
Med     L            6 20 45  2
        S           41 28 48  2
>
```

```
> tab.burden.3 <- xtabs(~InsL2+Res+Burden,
data=pdat)
> ftable(tab.burden.3)
         Burden  A  B  C  D
InsL2 Res
Hi    No         5  9 38  3
      Yes       26 11  5  0
Lo    No         4  5 13  0
      Yes        8  3  1  0
Med   No        11  1 15  1
      Yes        6  6  2  0
Nil   No        11 24 65  7
      Yes       14 19 16  1
## Making a new data table by merging D to
C
         Burden  A  B  C
InsL2 Res
Hi    No         5  9 41
      Yes       26 11  5
Lo    No         4  5 13
      Yes        8  3  1
```

```
> tab.burden.4 <- xtabs(~AvgCost+ InsL2+
Burden, data=pdat)
> ftable(tab.burden.4)
              Burden  A  B  C  D
AvgCost InsL2
Hi      Hi            3  4 20  2
        Lo            0  2  6  0
        Med           1  1  7  1
        Nil           4  7 14  4
Lo      Hi           13  3  4  0
        Lo            4  1  0  0
        Med           8  3  2  0
        Nil           5  9  9  1
Med     Hi           15 13 19  1
        Lo            8  5  8  0
        Med           8  3  8  0
        Nil          16 27 58  3
## Making new table by merging D to C
              Burden  A  B  C
AvgCost InsL2
Hi      Hi            3  4 22
```

©2014 Q.H. Vuong

```
Med    No          11  1 16
       Yes          6  6  2
Nil    No          11 24 72
       Yes         14 19 17
```

```
         Lo             0  2  6
         Med            1  1  8
         Nil            4  7 18
Lo       Hi            13  3  4
         Lo             4  1  0
         Med            8  3  2
         Nil            5  9 10
Med      Hi            15 13 19
         Lo             8  5  8
         Med            8  3  8
         Nil           16 27 61
## Making even more table by merging rows
Lo and Nil (of InsL2) from the new table
(preceding)
               Burden  A  B  C
AvgCost InsL2
Hi       Hi             3  4 22
         Med            1  1  8
         Neg            4  9 24
Lo       Hi            13  3  4
         Med            8  3  2
         Neg            9 10 10
Med      Hi            15 13 19
         Med            8  3  8
         Neg           24 32 69
```

```
> tab.env.1 <- xtabs(~Ill2+SES+EnvL,
data=pdat)
> ftable(tab.env.1)
           EnvL Hi Lo Med Nil
Ill2  SES
Bad   Hi         1  2   8   1
      Lo         0 11   1  13
      Med       14 41  21  35
Emerg Hi         2  1   1   2
      Lo         3  4   0   3
      Med        5 18   9  28
Light Hi         7  1   6   2
      Lo         4  4   7   5
      Med       24 19  15  12
##env1.txt
```

```
> tab.env.2 <- xtabs(~Ill2+IncRank+EnvL,
data=pdat)
> ftable(tab.env.2)
               EnvL Hi Lo Med Nil
Ill2  IncRank
Bad   Hi             0  0   0   1
      Lo             8 43  17  43
      Mid            7 11  13   5
Emerg Hi             1  0   0   1
      Lo             9 20   8  27
      Mid            0  3   2   5
Light Hi             3  1   1   0
      Lo            16 13  15  11
      Mid           16 10  12   8
## making new data table by merging rows
Hi and Mid of tab.env.2
               EnvL Hi Lo Med Nil
Ill2  IncRank
Bad   HM             7 11  13   6
      Lo             8 43  17  43
Emerg HM             1  3   2   6
      Lo             9 20   8  27
Light Hi            19 11  13   8
      Lo            16 13  15  11
## env2.txt
```

```
> tab.end.1 <- xtabs(~Ill2+EnvL+End,
data=pdat)
> ftable(tab.end.1)
            End  A  B  C  D
Ill2  EnvL
Bad   Hi         8  6  1  0
      Lo         6 46  2  0
      Med       10 16  4  0
      Nil        8 32  8  1
Emerg Hi         2  3  5  0
      Lo         2 11  9  1
      Med        3  5  2  0
      Nil        4 14 13  2
```

```
> tab.end.2 <- xtabs(~Ill2+AvgCost+End,
data=pdat)
> ftable(tab.end.2)
               End  A  B  C  D
Ill2  AvgCost
Bad   Hi            3 33  6  0
      Lo           12  8  1  0
      Med          17 59  8  1
Emerg Hi            0  8 12  2
      Lo            5  1  1  0
      Med           6 24 16  1
Light Hi            3  8  1  0
      Lo           21 12  1  0
```

©2014 Q.H. Vuong

```
Light Hi        27  8  0  0
      Lo         9 14  1  0
      Med       13 15  0  0
      Nil        9  9  1  0
## make a new table by merging C and D
           End  A  B  C
Ill2  EnvL
Bad   Hi         8  6  1
      Lo         6 46  2
      Med       10 16  4
      Nil        8 32  9
Emerg Hi         2  3  5
      Lo         2 11  10
      Med        3  5  2
      Nil        4 14 15
Light Hi        27  8  0
      Lo         9 14  1
      Med       13 15  0
      Nil        9  9  1
```

```
      Med         34 26  0  0
##
## make a new table by merging C and D
above
              End  A  B  C
Ill2  AvgCost
Bad   Hi           3 33  6
      Med         17 59  9
      Lo          12  8  1
Emerg Hi           0  8 14
      Med          6 24 17
      Lo           5  1  1
Light Hi           3  8  1
      Med         34 26  0
      Lo          21 12  1
>
## end2.txt
```

```
> tab.end.3 <- xtabs(~Ill2+Stay+End,
data=pdat)
> ftable(tab.end.3)
           End  A  B  C  D
Ill2  Stay
Bad   L         15 48  8  0
      S         17 52  7  1
Emerg L          7 14 17  2
      S          4 19 12  1
Light L          1  3  0  0
      S         57 43  2  0
>
## make a new table by merging C and D
above
           End  A  B  C
Ill2  Stay
Bad   L         15 48  8
      S         17 52  8
Emerg L          7 14 19
      S          4 19 13
Light L          1  3  0
      S         57 43  2
```

```
> attach(pdat)
> tab.resinsburden.1 <-
xtabs(~Res+Insured+Burden, data=pdat)
> ftable(tab.resinsburden.1)
            Burden  A  B  C  D
Res Insured
No  No              9 23 64  7
    Yes            22 16 67  4
Yes No             14 18 13  1
    Yes            40 21 11  0
>
## make a new table by merging C and D
above into a new C column
            Burden  A  B  C
Resident Insured
Nonres   No         9 23 71
         Yes       22 16 71
Res      No        14 18 14
         Yes       40 21 11
##burden1.txt
```

Plots

```
> plot(Days,Spent)
```

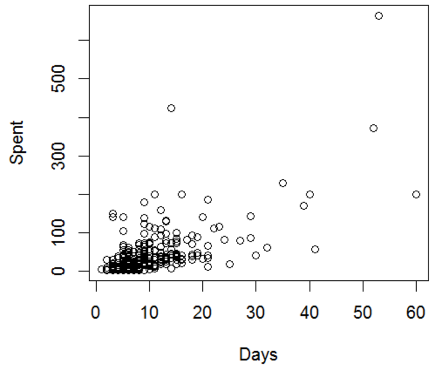


```
> plot(Days,Dcost)
```

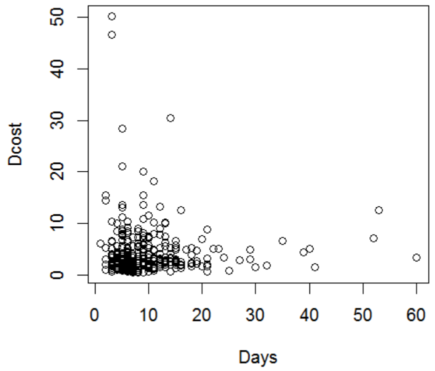


```
> plot(Age,Days)
```

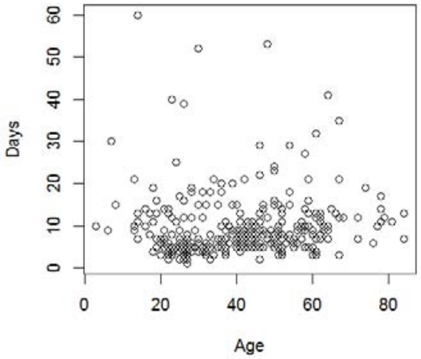


```
> plot(Burden,InsL2)
```

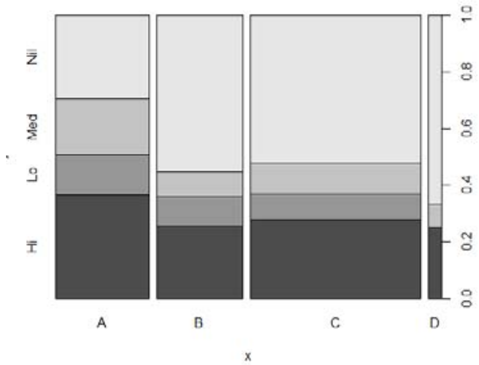


```
> plot(Res, Senv)
```

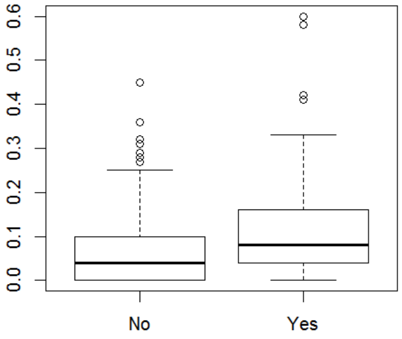


```
> plot(Ill2, Senv)
```

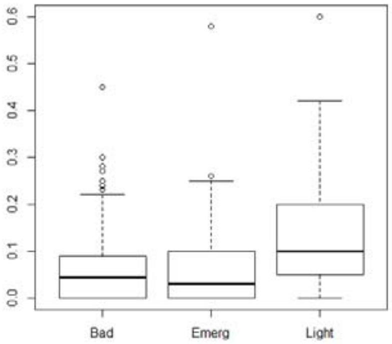


```
> plot(Burden,Income)
```

```
> plot(Burden,Spent)
```

©2014 Q.H. Vuong

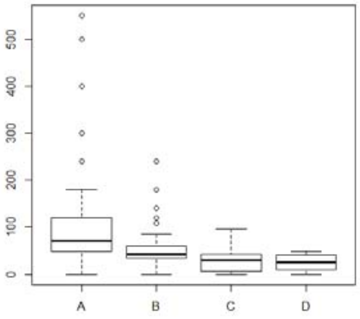

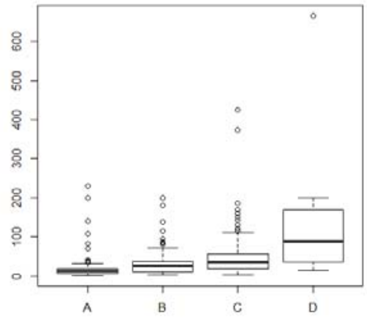


```
> boxplot(Income, Spent)
```

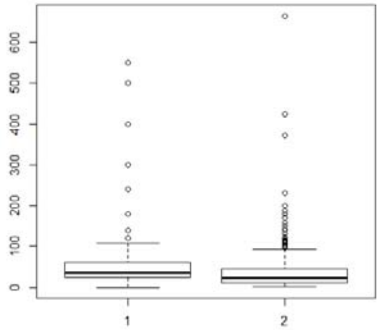


```
> plot(End,Spent)
```

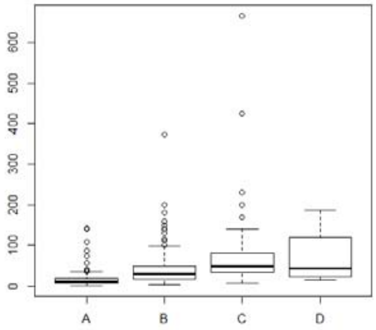

> plot(End,Dcost)

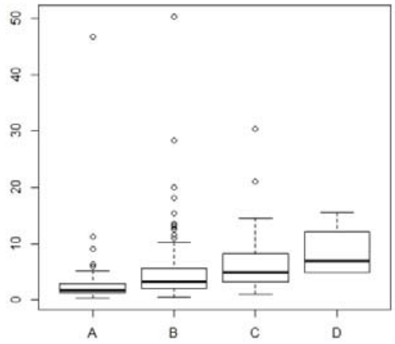


```
> library(scatterplot3d)
> scatterplot3d(Dcost,Days,Spent)
```

> plot(SES,Spent)

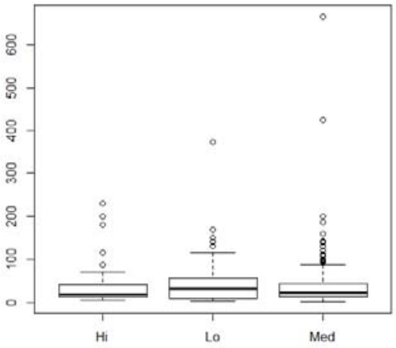


```
> plot(Res,Days)
```

©2014 Q.H. Vuong

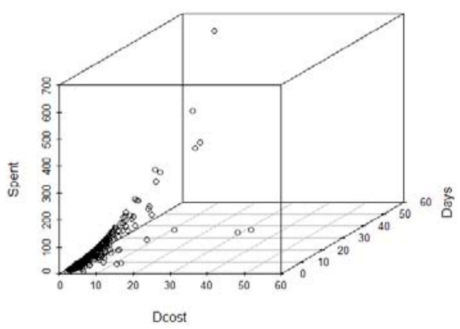

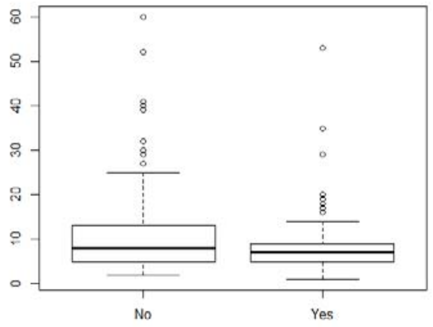


```
> install.packages("Rcmdr")
> library(Rcmdr)
> install.packages("rgl")
> scatter3d(Days,Dcost,Spent)
```

```
> r <- Days
> symbols(Dcost,Spent, circle=r,
inches=0.3, fg="white",bg="blue",main=
"Bubble plot", ylab="Money spent",
xlab="Daily cost")
> text(Dcost,Spent,ID,cex=0.6)
```

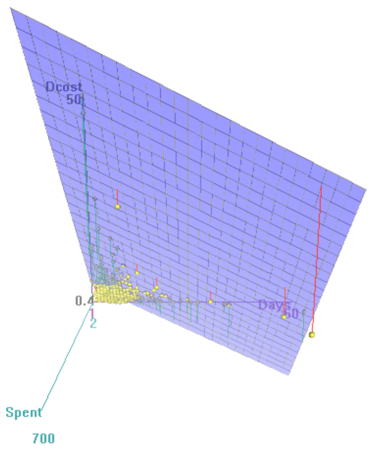

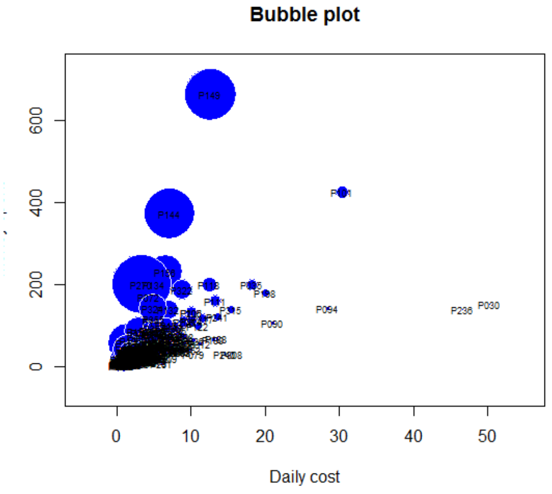


```
> r<- log(Days)
## This is to reduce the difference in
size of bubble
> symbols(Dcost,Spent, circle=r,
inches=0.3, fg="white", bg="blue",
main="Bubble plot",ylab="Money spent",
xlab="Daily cost")
```

## to insert ID value to the bubble, use this command: > text(Dcost,Spent,ID,cex=0.6)

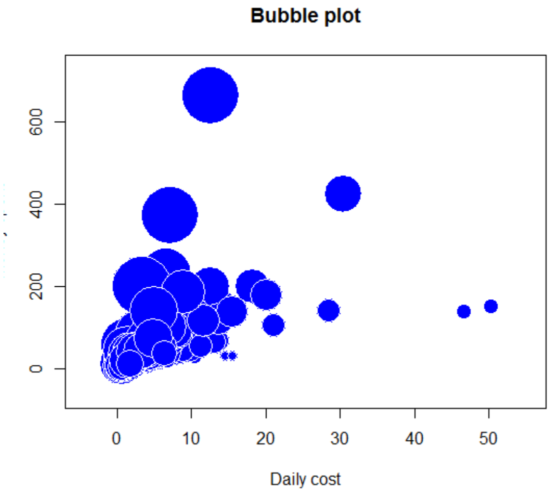

Actual modeling using P330.csv data set

```
## New data table by merging D to C
          Burden  A  B  C
InsL2 Res
Hi    No          5  9 41
      Yes        26 11  5
Lo    No          4  5 13
      Yes         8  3  1
Med   No         11  1 16
      Yes         6  6  2
Nil   No         11 24 72
      Yes        14 19 17
## burden3.txt
```

```
> burden3
  InsL2 Res  A  B  C
1    Hi  No  5  9 41
2    Hi Yes 26 11  5
3    Lo  No  4  5 13
4    Lo Yes  8  3  1
5   Med  No 11  1 16
6   Med Yes  6  6  2
7   Nil  No 11 24 72
8   Nil Yes 14 19 17
```

```
> burden3 <- read.table("c:/Dr.Vuong/PS/burden3.txt",  header=TRUE)
> contrasts(burden3$InsL2)=contr.treatment(levels(burden3$InsL2),base=1)
> contrasts(burden3$Res)=contr.treatment(levels(burden3$Res),base=2)
> fit.burden3=vglm(cbind(C,B,A)~InsL2+Res, data=burden3, family=multinomial)
> summary(fit.burden3)
Call:
vglm(formula = cbind(C, B, A) ~ InsL2 + Res, family = multinomial,
    data = burden3)
Pearson residuals:
  log(mu[,1]/mu[,3]) log(mu[,2]/mu[,3])
1            1.31636            0.71294
2           -1.47814           -0.38736
3            0.39104            0.39447
4           -0.58534           -0.35792
5           -0.45820           -1.90298
6            0.32869            2.15598
7           -0.91744            0.12712
8            1.30359           -0.33535
Coefficients:
               Estimate Std. Error   z value
(Intercept):1 -0.927882    0.31694 -2.927598
(Intercept):2 -0.646550    0.31298 -2.065795
InsL2Lo:1     -0.520450    0.50954 -1.021403
InsL2Lo:2     -0.046771    0.54501 -0.085817
InsL2Med:1    -0.671408    0.46192 -1.453511
InsL2Med:2    -0.545827    0.53975 -1.011267
InsL2Nil:1     0.714368    0.35900  1.989878
InsL2Nil:2     0.936850    0.38412  2.438961
ResNo:1        2.338077    0.32361  7.224900
ResNo:2        0.580802    0.33054  1.757154
Number of linear predictors:  2
Names of linear predictors: log(mu[,1]/mu[,3]), log(mu[,2]/mu[,3])
Dispersion Parameter for multinomial family:   1
Residual deviance: 17.31636 on 6 degrees of freedom
Log-likelihood: -35.42623 on 6 degrees of freedom
Number of iterations: 5
z-value       X^2    p-value
-2.9276       8.5708 0.0034
-2.0658       4.2675 0.0388
-1.0214       1.0433 0.3071
-0.0858       0.0074 0.9316
-1.4535       2.1127 0.1461
-1.0113       1.0227 0.3119
1.9899        3.9596 0.0466
2.4390        5.9485 0.0147
7.2249        52.1992 0.0000
```

1.7572	3.0876	0.0789

Burden3 Results

	Intercept	Resident	InsL2		
		No	Lo	Med	Nil
	β0	β1	β2	β3	β4
logit(C|A)	-0.9279***<br>(-2.9276)	2.3381***<br>(7.2249)	-0.52045<br>(-1.0214)	-0.6714<br>(-1.4535)	0.7144**<br>(1.9899)
Logit(B|A)	-0.6466**<br>(-2.0658)	0.5808*<br>(1.7572)	-0.0468<br>(-0.0858)	-0.5458<br>(-1.0113)	0.9368**<br>(2.4390)
Baseline = no financial burden at all; z-values in parentheses; (***,**,*) denote coefficients significant at 1, 5 and 10% respectively.					

Explanations of Burden3 results:

This is to model the probability of falling into burden cat C (and B) vs. cat A, depending on whether a patient is "non-resident" and "uninsured". The results show very clear. Both burden of categories C (distressed) and B(somewhat burdensome) shows effects being non-resident and having no insurance. In other words, having no insurance and being non-resident increases the log-odds of falling into type C or B of burden.

$$\log\left(\frac{\hat{\pi}_C}{\hat{\pi}_A}\right) = -0.9279 + 2.3881 NonRes - 0.5204 InsLow - 0.6714 InsMed + 0.7144 InsNil$$

$$\log\left(\frac{\hat{\pi}_B}{\hat{\pi}_A}\right) = -0.6466 + 0.5808 NonRes - 0.0468 InsLow - 0.5458 InsMed + 0.9368 InsNil$$

Estimating the probability that a nonresident patient falling into debt having no insurance $\hat{\pi}_C$:

$$\hat{\pi}_C = \frac{e^{-0.9279+2.3881+0.7144}}{1 + e^{-0.9279+2.3881+0.7144} + e^{-0.6466+0.5808+0.9368}} = 0.6945$$

And, the probability that a nonresident patient falling into some kind of adverse effect (but not indebtedness) having no insurance $\hat{\pi}_C$:

$$\hat{\pi}_B = \frac{e^{-0.6466+0.5808+0.9368}}{1 + e^{-0.9279+2.3881+0.7144} + e^{-0.6466+0.5808+0.9368}} = 0.2534$$

Only 5.2% will not be adversely affected at all if hospitalized without insurance while being nonresident.

Burden 4 modeling

```
## New data table
## Making new table by merging D to C
               Burden  A  B  C
AvgCost InsL2
Hi      Hi             3  4 22
        Lo             0  2  6
        Med            1  1  8
        Nil            4  7 18
Lo      Hi            13  3  4
        Lo             4  1  0
        Med            8  3  2
        Nil            5  9 10
Med     Hi            15 13 19
        Lo             8  5  8
        Med            8  3  8
        Nil           16 27 61
## burden4a.txt
```

```
## Making even more table by merging rows
Lo and Nil (of InsL2) from the new table
(preceding)
               Burden  A  B  C
AvgCost InsL2
Hi      Hi             3  4 22
        Med            1  1  8
        Neg            4  9 24
Lo      Hi            13  3  4
        Med            8  3  2
        Neg            9 10 10
Med     Hi            15 13 19
        Med            8  3  8
        Neg           24 32 69
##burden4.txt
```

```
> burden4 <- read.table("c:/Dr.Vuong/PS/burden4.txt",  header=TRUE)
> burden4
  AvgCost InsL2  A  B  C
1  HiCost    Hi  3  4 22
2  HiCost   Med  1  1  8
3  HiCost   Neg  4  9 24
4 LowCost    Hi 13  3  4
5 LowCost   Med  8  3  2
6 LowCost   Neg  9 10 10
7 MedCost    Hi 15 13 19
8 MedCost   Med  8  3  8
9 MedCost   Neg 24 32 69
> contrasts(burden4$AvgCost)=contr.treatment(levels(burden4$AvgCost),base=2)
> contrasts(burden4$InsL2)=contr.treatment(levels(burden4$InsL2),base=1)
> install.packages("VGAM")
> library(VGAM)
> fit.burden4=vglm(cbind(C,B,A)~AvgCost+InsL2, data=burden4, family=multinomial)
> fit.burden4=vglm(cbind(C,B,A)~AvgCost+InsL2, data=burden4, family=multinomial)
> summary(fit.burden4)
Call:
vglm(formula = cbind(C, B, A) ~ AvgCost + InsL2, family = multinomial,
    data = burden4)
Pearson residuals:
  log(mu[,1]/mu[,3]) log(mu[,2]/mu[,3])
1            0.80302          -0.282849
2            0.70086          -0.161683
3           -1.28558           0.065725
4           -0.31232          -0.823560
5           -0.43045           0.532126
6            0.53929           0.394384
7           -0.33649           0.871469
8           -0.20118          -0.251428
9            0.26079          -0.395385
Coefficients:
                  Estimate Std. Error  z value
(Intercept):1     -0.94615    0.37790 -2.50372
(Intercept):2     -0.92833    0.39631 -2.34241
AvgCostHiCost:1    2.56937    0.49636  5.17637
AvgCostHiCost:2    1.21280    0.54854  2.21096
AvgCostMedCost:1   1.21576    0.36412  3.33893
AvgCostMedCost:2   0.50195    0.38078  1.31821
InsL2Med:1        -0.14362    0.44422 -0.32331
InsL2Med:2        -0.36563    0.53947 -0.67776
InsL2Neg:1         0.76424    0.32306  2.36563
```

```
InsL2Neg:2         0.79270    0.36597  2.16604
Number of linear predictors:  2
Names of linear predictors: log(mu[,1]/mu[,3]), log(mu[,2]/mu[,3])
Dispersion Parameter for multinomial family:   1
Residual deviance: 5.72308 on 8 degrees of freedom
Log-likelihood: -32.19016 on 8 degrees of freedom
Number of iterations: 4
## Estimated by Quan Hoang Vuong, 5:22PM Oct 12, 2014
## z^2=Wald chi-square, df=1. Use excel chidist(z^2,1) to produce p-value.
z-value      X^2    p-value
-2.5037      6.2686 0.0123
-2.3424      5.4869 0.0192
5.1764       26.7948 0.0000
2.2110       4.8883 0.0270
3.3389       11.1485 0.0008
1.3182       1.7377 0.1874
-0.3233      0.1045 0.7465
-0.6778      0.4594 0.4979
2.3656       5.5962 0.0180
2.1660       4.6917 0.0303
```

Burden 4 estimation results (tabulated)

		AvgCost		Insurance Level	
		HiCost	MedCost	Med	Neg
	β0	β1	β2	β3	β4
logit(C|A)	-0.9462**<br>(-2.5037	2.5694***<br>(5.1764)	1.2158***<br>(3.3389)	-0.1436<br>(-0.3233)	0.7642**<br>(2.3656)
Logit(B|A)	-0.9283**<br>(-2.3424)	1.2128**<br>(2.2110)	0.5020<br>(1.3182)	-0.3656<br>(-0.6778)	0.7927**<br>(2.1660)
z-value in brackets. Baseline category: No financial burden after staying in hospital. (***) signif at 1%; (**) signif at 5%; (*) signif at 10%.					

Burden 4 explanations:

$$\log\left(\frac{\hat{\pi}_C}{\hat{\pi}_A}\right) = -0.9462 + 2.5694HiCost + 1.2158MedCost - 0.1436InsMed + 0.7642InsNeg$$

$$\log\left(\frac{\hat{\pi}_B}{\hat{\pi}_A}\right) = -0.9283 + 1.2128HiCost + 0.5020MedCost - 0.3656InsMed + 0.7927InsNeg$$

Estimating the probability that a patient falling into debt having negligible insurance benefits while paying high health care cost $\hat{\pi}_C$:

$$\hat{\pi}_C = \frac{e^{-0.9462+2.5694+0.7642}}{1 + e^{-0.9462+2.5694+0.7642} + e^{-0.9283+1.2128+0.7927}} = 0.6912$$

And, the probability that a patient falling into some kind of adverse effect (but not indebtedness) having negligible or no insurance $\hat{\pi}_B$ while paying higher cost of services:

$$\hat{\pi}_C = \frac{e^{-0.9283+1.2128+0.7927}}{1 + e^{-0.9462+2.5694+0.7642} + e^{-0.9283+1.2128+0.7927}} = 0.2645$$

Only 4.43% will not be adversely affected at all if hospitalized without insurance while paying higher costs.

End3 modeling

```
## make a new table by merging C and D
           End  A  B  C
Ill2  Stay
Bad   L        15 48  8
      S        17 52  8
Emerg L         7 14 19
      S         4 19 13
Light L         1  3  0
      S        57 43  2
```

```
## end3.txt
Ill2  Stay      A  B  C
Bad   L        15 48  8
Bad   S        17 52  8
Emerg L         7 14 19
Emerg S         4 19 13
Light L         1  3  0
Light S        57 43  2
```

```
> end3 <- read.table("c:/Dr.Vuong/PS/end3.txt", header=TRUE)
> contrasts(end3$Stay)=contr.treatment(levels(end3$Stay),base=1)
> contrasts(end3$Ill2)=contr.treatment(levels(end3$Ill2),base=3)
> fit.end3=vglm(cbind(C,B,A)~Ill2+Stay, data=end3, family=multinomial)
> summary(fit.end3)
Call:
vglm(formula = cbind(C, B, A) ~ Ill2 + Stay, family = multinomial,
    data = end3)
Pearson residuals:
  log(mu[,1]/mu[,3]) log(mu[,2]/mu[,3])
1          -0.202023            0.21032
2           0.227046           -0.20546
3          -0.109097           -0.86502
4           0.081518            0.87148
5          -0.182055            1.31192
6           0.047590           -0.25895
Coefficients:
               Estimate Std. Error   z value
(Intercept):1 -3.109622    0.82728 -3.758842
(Intercept):2 -0.260721    0.37613 -0.693170
Ill2Bad:1      2.548144    0.80743  3.155877
Ill2Bad:2      1.384345    0.31856  4.345600
Ill2Emerg:1    4.302720    0.82594  5.209489
Ill2Emerg:2    1.344241    0.42874  3.135313
StayS:1       -0.269351    0.42885 -0.628072
StayS:2        0.030045    0.33258  0.090339
Number of linear predictors:  2
Names of linear predictors: log(mu[,1]/mu[,3]), log(mu[,2]/mu[,3])
Dispersion Parameter for multinomial family:   1
Residual deviance: 3.62253 on 4 degrees of freedom
Log-likelihood: -21.96001 on 4 degrees of freedom
Number of iterations: 4
### Estimated by Quan Hoang Vuong 6:14PM Oct 12, 2014.
## z^2=Wald chi-square, df=1. Use excel chidist(z^2,1) to produce p-value.
z-value       X^2 Wald      p-value
-3.758842     14.12889318   0.000170702
-0.69317      0.480484649   0.488202874
3.155877      9.959559639   0.001600163
4.3456        18.88423936   1.38895E-05
5.209489      27.13877564   1.89361E-07
3.135313      9.830187608   0.001716708
-0.628072     0.394474437   0.529956775
0.090339      0.008161135   0.928017829
```

```
> burden1 <- read.table("c:/Dr.Vuong/PS/burden1.txt", header=TRUE)
> contrasts(burden1$Resident)=contr.treatment(levels(burden1$Resident),base=2)
```

```
> contrasts(burden1$Insured)=contr.treatment(levels(burden1$Insured),base=2)
> fit.burden1=vglm(cbind(C,B,A)~Resident+Insured, data=burden1, family=multinomial)
> summary(fit.burden1)
Call:
vglm(formula = cbind(C, B, A) ~ Resident + Insured, family = multinomial,
    data = burden1)
Pearson residuals:
  log(mu[,1]/mu[,3]) log(mu[,2]/mu[,3])
1           -0.30824            0.35222
2            0.29383           -0.40647
3            0.56157           -0.42728
4           -0.54440            0.42962
Coefficients:
                 Estimate Std. Error z value
(Intercept):1    -1.12387    0.27381 -4.1046
(Intercept):2    -0.73490    0.25157 -2.9213
ResidentNonres:1  2.26280    0.31777  7.1209
ResidentNonres:2  0.52219    0.32639  1.5999
InsuredNo:1       0.96518    0.31530  3.0612
InsuredNo:2       1.07767    0.33488  3.2181
Number of linear predictors:  2
Names of linear predictors: log(mu[,1]/mu[,3]), log(mu[,2]/mu[,3])
Dispersion Parameter for multinomial family:   1
Residual deviance: 1.45223 on 2 degrees of freedom
Log-likelihood: -17.92251 on 2 degrees of freedom
Number of iterations: 3
z-value      X^2 Wald     p-value
-4.1046      16.8477      4.05015E-05
-2.9213      8.5340       0.00348574
7.1209       50.7072       1.07225E-12
1.5999       2.5597        0.109620799
3.0612       9.3709       0.002204518
3.2181       10.3562      0.001290428
## Estimated by Quan Hoang Vuong 6:36PM Oct 12, 2014.
```

Estimation results for Burden1

	Intercept	Resident	Insured
		No	No
	β0	β1	β2
logit(C|A)	-1.1239***<br>[0.2738]<br>(-4.1046)	2.2628***<br>[0.3178]<br>(7.1209)	0.9652***<br>[0.3153]<br>(3.0612)
Logit(B|A)	-0.7349***<br>[0.2516]<br>(-2.9213)	0.5222<br>[0.3264]<br>(1.5999)	1.0777***<br>[0.3349]<br>(3.2181)
Baseline = no financial burden at all; (s.e) and z-values in parentheses [] and (); (***,**,*) denote coefficients significant at 1, 5 and 10% respectively.			

Burden 1 explanations:

$$\log\left(\frac{\hat{\pi}_C}{\hat{\pi}_A}\right) = -1.1239 + 2.2628 NonRes + 0.9652 Uninsured$$

$$\log\left(\frac{\hat{\pi}_B}{\hat{\pi}_A}\right) = -0.7349 + 0.5222 NonRes + 1.0777 Uninsured$$

Estimating the probability that a patient falls into debt if that patient is nonresident and has no insurance (or costs are not eligible for reimbursement under the policy) $\hat{\pi}_C$:

$$\hat{\pi}_C = \frac{e^{-1.1239+2.2628+0.9652}}{1 + e^{-1.1239+2.2628+0.9652} + e^{-0.7349+0.5222+1.0777}} = 0.7084$$

And, the probability that a patient falling into some kind of adverse effect (but not indebtedness) having negligible or no insurance $\hat{\pi}_B$ while being non-resident:

$$\hat{\pi}_B = \frac{e^{-0.9283+1.2128+0.7927}}{1 + e^{-0.9462+2.5694+0.7642} + e^{-0.9283+1.2128+0.7927}} = 0.2052$$

Only 8.64% will not be adversely affected at all if hospitalized without insurance while being non-resident.

Modeling end2

```
> end2 <- read.table("c:/Dr.Vuong/PS/end2.txt", header=TRUE)
> contrasts(end2$Ill2)=contr.treatment(levels(end2$Ill2),base=3)
> contrasts(end2$AvgCost)=contr.treatment(levels(end2$AvgCost),base=2)
> fit.end2=vglm(cbind(C,B,A)~Ill2+AvgCost, data=end2, family=multinomial)
> summary(fit.end2)
Call:
vglm(formula = cbind(C, B, A) ~ Ill2 + AvgCost, family = multinomial,
    data = end2)
Pearson residuals:
  log(mu[,1]/mu[,3]) log(mu[,2]/mu[,3])
1           -0.58294            0.25702
2            0.74548            0.30809
3           -0.18330           -0.92771
4            0.88234            0.36474
5            0.14071            0.71798
6           -1.01376           -1.51948
7            0.31871           -0.55646
8           -1.14364           -0.75963
9            1.53501            1.49831
Coefficients:
              Estimate Std. Error z value
(Intercept):1  -4.5965    0.93044 -4.9401
(Intercept):2  -1.1231    0.31481 -3.5675
Ill2Bad:1       2.3444    0.79443  2.9511
Ill2Bad:2       1.1452    0.29832  3.8388
Ill2Emerg:1     4.1486    0.81426  5.0950
Ill2Emerg:2     1.1074    0.41619  2.6608
AvgCostHi:1     3.2568    0.81224  4.0096
AvgCostHi:2     2.4116    0.52296  4.6114
AvgCostMed:1    1.2881    0.69507  1.8532
AvgCostMed:2    1.0800    0.32988  3.2739
Number of linear predictors:  2
Names of linear predictors: log(mu[,1]/mu[,3]), log(mu[,2]/mu[,3])
Dispersion Parameter for multinomial family:   1
Residual deviance: 14.36112 on 8 degrees of freedom
Log-likelihood: -32.1729 on 8 degrees of freedom
Number of iterations: 5
z-value      Wald   p-value
-4.9401      24.4046      7.80825E-07
-3.5675      12.7271      0.000360403
2.9511       8.7090       0.003166444
3.8388       14.7364      0.000123637
5.095        25.9590      3.48741E-07
2.6608       7.0799       0.007795525
4.0096       16.0769      6.08217E-05
4.6114       21.2650      3.99966E-06
1.8532       3.4344       0.063853711
3.2739       10.7184      0.001060741
```

End2 estimation results

		Illness		Average Cost of Services	
		Bad	Emergency	High	Medium
	β0	β1	β2	β3	β4
logit(C|A)	-4.5965***<br>(-4.9401)	2.3444***<br>(2.9511)	4.1486***<br>(5.0950)	3.2568***<br>(4.0096)	1.2881*<br>(1.8532)
Logit(B|A)	-1.1231***<br>(-3.5675)	1.1452***<br>(3.8388)	1.1074***<br>(2.6608)	2.4116***<br>(4.6114)	1.0800***<br>(3.2739)

z-value in brackets. Baseline category: Complete recovery after treatments. (***) signif at 1%; (**) signif at 5%; (*) signif at 10%.

$$\log\left(\frac{\hat{\pi}_C}{\hat{\pi}_A}\right) = -4.5965 + 2.3444Bad + 4.1486Emergency + 3.2568HighCost + 1.2881MedCost$$

$$\log\left(\frac{\hat{\pi}_B}{\hat{\pi}_A}\right) = -1.1231 + 1.1452Bad + 1.1074Emergency + 2.4116HighCost + 1.08MedCost$$

Computing probabilities

Estimating the probability that a patient quits while in bad condition and see high cost of treatments $\hat{\pi}_C$:

$$\hat{\pi}_C = \frac{e^{-4.5965+4.1486+3.2568}}{1 + e^{-4.5965+4.1486+3.2568} + e^{-1.1231+1.1074+2.4116}} = 0.5805$$

And, the probability that a patient can be partially cured $\hat{\pi}_B$ while suffering high cost of services:

$$\hat{\pi}_B = \frac{e^{-1.1231+1.1074+2.4116}}{1 + e^{-4.5965+4.1486+3.2568} + e^{-1.1231+1.1074+2.4116}} = 0.3845$$

Only 3.5% can recover fully after treatments, paying higher costs and in emergency.

Modeling env2 estimation

```
> env2 <- read.table("c:/Dr.Vuong/PS/env2.txt", header=TRUE)
> contrasts(env2$Ill2)=contr.treatment(levels(end2$Ill2),base=3)
> contrasts(env2$IncRank)=contr.treatment(levels(env2$IncRank),base=2)
> fit.env2=vglm(cbind(Hi,Med,Nil)~Ill2+IncRank, data=env2, family=multinomial)
> summary(fit.env2)
Call:
vglm(formula = cbind(Hi, Med, Nil) ~ Ill2 + IncRank, family = multinomial,
    data = env2)
Pearson residuals:
  log(mu[,1]/mu[,3]) log(mu[,2]/mu[,3])
1         7.7762e-01         3.9617e-01
2        -5.3359e-01        -2.4858e-01
3        -1.1974e+00        -7.4790e-01
4         6.1379e-01         3.7416e-01
5         1.1464e-16        -1.8389e-16
6         0.0000e+00         0.0000e+00
Coefficients:
              Estimate Std. Error  z value
(Intercept):1  0.50791    0.58132  0.87372
(Intercept):2  0.61038    0.51284  1.19020
Ill2Bad:1     -1.76845    0.45605 -3.87777
Ill2Bad:2     -1.07513    0.41346 -2.60034
Ill2Emerg:1   -1.48041    0.48507 -3.05198
Ill2Emerg:2   -1.46054    0.48799 -2.99297
IncRankHi:1   -0.50791    0.66573 -0.76294
IncRankHi:2   -0.98987    0.62654 -1.57989
IncRankL:1    -0.91337    0.48349 -1.88913
IncRankL:2    -1.08039    0.39328 -2.74711
Number of linear predictors:  2
```

```
Names of linear predictors: log(mu[,1]/mu[,3]), log(mu[,2]/mu[,3])
Dispersion Parameter for multinomial family:   1
Residual deviance: 3.80586 on 2 degrees of freedom
Log-likelihood: -24.10649 on 2 degrees of freedom
Number of iterations: 4
##
z-value       X^2 Wald      p-value
0.87372       0.763386638   0.382270758
1.1902        1.41657604    0.23396794
-3.87777      15.03710017   0.000105418
-2.60034      6.761768116   0.009313144
-3.05198      9.31458192    0.002273373
-2.99297      8.957869421   0.002762769
-0.76294      0.582077444   0.445499185
-1.57989      2.496052412   0.114132097
-1.88913      3.568812157   0.058874421
-2.74711      7.546613352   0.006012297
```

Estimation results following env2:

		Illness		Income Rank	
		Bad	Emergency	High	Low
	β0	β1	β2	β3	β4
logit(HiPay|Neg)	0.5079	-1.7684***	-1.4804***	-0.5079	-0.9134*
	[0.5813]	[0.4560]	[0.4851]	[0.6657]	[0.4835]
	(0.8737)	(-3.8778)	(-3.0520)	(-0.7629)	(-1.8891)
Logit(MedPay|Neg)	0.6104	-1.0751***	-1.4605***	-0.9899	-1.0804***
	[0.5128]	0.41346	[0.4880]	[0.6265]	[0.3933]
	(1.1902)	(-2.6003)	(-2.9930)	(-1.5799)	(-2.7471)
z-value in brackets. Baseline category: Pay negligible "extra envelop" amount; (***) signif at 1%; (**) significant at 5%; (*) signif at 10%.					

$$\log\left(\frac{\hat{\pi}_{HiPay}}{\hat{\pi}_{NegPay}}\right) = +0.5079 - 1.7684Bad - 1.4804Emerg - 0.5079HiInc - 0.9134LowInc$$

$$\log\left(\frac{\hat{\pi}_{MedPay}}{\hat{\pi}_{NegPay}}\right) = +0.6104 - 1.0751Bad - 1.4605Emerg - 0.9899HiInc - 1.0804LowInc$$

Computing probabilities

Estimating the probability that a patient in emergency and low-income pays high for envelop: $\hat{\pi}_{HiPay}$:

$$\hat{\pi}_{HiPay} = \frac{e^{+0.5079-1.4804-0.9134}}{1+e^{+0.5079-1.4804-0.9134}+e^{+0.6104-1.4605-1.0804}} = 0.1170$$

And, the probability that a patient can be partially cured $\hat{\pi}_B$ while suffering high cost of services:

$$\hat{\pi}_{MedPay} = \frac{e^{+0.6104-1.4605-1.0804}}{1+e^{+0.5079-1.4804-0.9134}+e^{+0.6104-1.4605-1.0804}} = 0.1119$$

Approx. 77.12% pays "extra envelop" of negligible amount if they are in emergency if they belong to low-income group of patients.

```
> env1 <- read.table("c:/Dr.Vuong/PS/env1.txt", header=TRUE)
> contrasts(env1$Ill2)=contr.treatment(levels(env1$Ill2),base=3)
> contrasts(env1$SES)=contr.treatment(levels(env1$SES),base=3)
> fit.env1=vglm(cbind(H,M,L,N)~Ill2+SES, data=env1, family=multinomial)
> summary(fit.env1)
Call:
vglm(formula = cbind(H, M, L, N) ~ Ill2 + SES, family = multinomial,
    data = env1)
Pearson residuals:
  log(mu[,1]/mu[,4]) log(mu[,2]/mu[,4]) log(mu[,3]/mu[,4])
1           -0.40367            1.31586           0.375756
2            0.80279            0.05911          -0.134869
3           -1.36774           -1.40518           0.179137
4            0.35314           -0.84921           0.015322
5           -1.10194            0.59368          -0.328760
6            2.80909           -0.70429           0.715838
7           -0.16708           -0.79482          -0.448057
8            0.10476           -0.41011           0.569434
9           -0.31651            1.55762          -0.775742
Coefficients:
               Estimate Std. Error  z value
(Intercept):1 -0.120238    0.49190 -0.24444
(Intercept):2 -0.302587    0.47768 -0.63344
(Intercept):3  0.106692    0.40410  0.26402
Ill2Bad:1     -1.801430    0.41606 -4.32972
Ill2Bad:2     -0.855584    0.38700 -2.21083
Ill2Bad:3     -0.153716    0.36627 -0.41968
Ill2Emerg:1   -1.847079    0.46800 -3.94672
Ill2Emerg:2   -1.593828    0.47972 -3.32241
Ill2Emerg:3   -0.620190    0.41261 -1.50309
SESA:1         1.845567    0.73011  2.52779
SESA:2         2.122548    0.67702  3.13511
SESA:3        -0.089649    0.74438 -0.12043
SESB:1         0.762756    0.49963  1.52664
SESB:2         0.604496    0.46691  1.29467
SESB:3         0.193766    0.35930  0.53929
Number of linear predictors:  3
Names of linear predictors: log(mu[,1]/mu[,4]), log(mu[,2]/mu[,4]), log(mu[,3]/mu[,4])
Dispersion Parameter for multinomial family:   1
Residual deviance: 22.41464 on 12 degrees of freedom
Log-likelihood: -49.62314 on 12 degrees of freedom
Number of iterations: 4
>
z-value      X^2 Wald     p-value
-0.24444     0.059750914  0.806890066
-0.63344     0.401246234  0.526446351
0.26402      0.06970656   0.791764508
-4.32972     18.74647528  1.49299E-05
-2.21083     4.887769289  0.027047614
-0.41968     0.176131302  0.674719238
-3.94672     15.57659876  7.92291E-05
-3.32241     11.03840821  0.000892435
-1.50309     2.259279548  0.13281589
2.52779      6.389722284  0.011478299
3.13511      9.828914712  0.001717897
-0.12043     0.014503385  0.904142528
1.52664      2.33062969   0.126850595
1.29467      1.676170409  0.195434204
0.53929      0.290833704  0.58968677
```

©2014 Q.H. Vuong

Examples (for consulting R codes) - old data set P205

```
             Burden  A  D
AvgCost InsL
Hi      A            0  4
        B           11 37
Med     A            1  6
        B           50 67
Lo      A            1  0
        B           20  8

> S=factor(rep(c("Hi","Mid","Lo"),c(2,2,2)))
> Shi=(S=="Hi")
> Smid=(S=="Mid")
> B=factor(rep(c("B","A"),3))
> Bins=(B=="B")
> y=c(37,4,67,6,8,0)
> n=c(37,4,67,6,8,0)+c(11,0,50,1,20,1)
> count=cbind(y,n-y)
> result=glm(count~Bins+Shi+Smid, family=binomial("logit"))
> summary(result)
Call:
glm(formula = count ~ Bins + Shi + Smid, family = binomial("logit"))

Deviance Residuals:
       1         2         3         4         5         6
-0.10685   0.80207  -0.04571   0.24851   0.23496  -1.26995

Coefficients:
            Estimate Std. Error z value Pr(>|z|)
(Intercept)   0.2149     0.8861   0.243  0.80834
BinsTRUE     -1.2302     0.8165  -1.507  0.13187
ShiTRUE       2.2651     0.5399   4.196 2.72e-05 ***
SmidTRUE      1.3165     0.4570   2.881  0.00397 **
---
Signif. codes:  0 '***' 0.001 '**' 0.01 '*' 0.05 '.' 0.1 ' ' 1

(Dispersion parameter for binomial family taken to be 1)
    Null deviance: 26.0894  on 5  degrees of freedom
Residual deviance:  2.3866  on 2  degrees of freedom
AIC: 25.029
Number of Fisher Scoring iterations: 4
```

***

As insurance coverage does not appear to bring in more explanatory power to the estimation, we cross it out, leaving only AvgCost as explanatory variables:

```
> result=glm(count~Shi+Smid, family=binomial("logit"))
> summary(result)

Call:
glm(formula = count ~ Shi + Smid, family = binomial("logit"))

Deviance Residuals:
      1        2        3        4        5        6
-0.2961   1.3789  -0.3524   1.5470   0.1162  -0.8035
```

```
Coefficients:
            Estimate Std. Error z value Pr(>|z|)
(Intercept)  -0.9651     0.4155  -2.323  0.02019 *
ShiTRUE       2.2808     0.5366   4.251 2.13e-05 ***
SmidTRUE      1.3237     0.4538   2.917  0.00353 **
---
Signif. codes:  0 '***' 0.001 '**' 0.01 '*' 0.05 '.' 0.1 ' ' 1

(Dispersion parameter for binomial family taken to be 1)

    Null deviance: 26.0894  on 5  degrees of freedom
Residual deviance:  5.1654  on 3  degrees of freedom
AIC: 25.808
```

***

```
             Burden  A  D
AvgCost Stay
Hi      L            4 12
        S            7 29
Med     L           15 34
        S           36 39
Lo      L            2  5
        S           19  3

> S=factor(rep(c("Hi","Mid","Lo"),c(2,2,2)))
> Shi=(S=="Hi")
> Smid=(S=="Mid")
> B=factor(rep(c("L","S"),3))
> Bstay=(B=="L")
> y=c(12,29,34,39,5,3)
> n=c(12,29,34,39,5,3)+c(4,7,15,36,2,19)
> count=cbind(y,n-y)
> result=glm(count~Bstay+Shi+Smid+Bstay*Shi+Bstay*Smid, family=binomial("logit"))
> summary(result)

Call:
glm(formula = count ~ Bstay + Shi + Smid + Bstay * Shi + Bstay *
    Smid, family = binomial("logit"))

Deviance Residuals:
[1]  0  0  0  0  0  0

Coefficients:
                   Estimate Std. Error z value Pr(>|z|)
(Intercept)         -1.8458     0.6213  -2.971  0.00297 **
BstayTRUE            2.7621     1.0421   2.651  0.00804 **
ShiTRUE              3.2672     0.7505   4.353 1.34e-05 ***
SmidTRUE             1.9259     0.6629   2.905  0.00367 **
BstayTRUE:ShiTRUE   -3.0849     1.2636  -2.441  0.01463 *
BstayTRUE:SmidTRUE  -2.0238     1.1115  -1.821  0.06864 .
---
Signif. codes:  0 '***' 0.001 '**' 0.01 '*' 0.05 '.' 0.1 ' ' 1

(Dispersion parameter for binomial family taken to be 1)
    Null deviance: 3.3146e+01  on 5  degrees of freedom
Residual deviance: 4.4408e-16  on 0  degrees of freedom
AIC: 32.674
Number of Fisher Scoring iterations: 3
```

***

```
        Burden  A  D
SES Res
Lo  No          2 35
    Yes         4  5
Med No         31 70
    Yes        29 12
Hi  No          7  0
    Yes        10  0

S=factor(rep(c("Lo","Med","Hi"),c(2,2,2)))
SSESlow=(S=="Lo")
SSESmed=(S=="Med")
B=factor(rep(c("No","Yes"),3))
Bres=(B=="No")
y=c(35,5,70,12,0,0)
n=c(35,5,70,12,0,0)+c(2,4,31,29,7,10)
count=cbind(y,n-y)
result=glm(count~Bres+SSESlow, family=binomial("logit"))
Call:
glm(formula = count ~ Bres + SSESlow, family = binomial("logit"))
Deviance Residuals:
      1        2        3        4        5        6
 0.5201  -0.5461   0.8015   1.0905  -3.8627  -2.2273
Coefficients:
            Estimate Std. Error z value Pr(>|z|)
(Intercept)  -1.2676     0.3188  -3.976 7.01e-05 ***
BresTRUE      1.9110     0.3631   5.262 1.42e-07 ***
SSESlowTRUE   1.8606     0.4996   3.724 0.000196 ***
---
Signif. codes:  0 '***' 0.001 '**' 0.01 '*' 0.05 '.' 0.1 ' ' 1

(Dispersion parameter for binomial family taken to be 1)
    Null deviance: 74.669  on 5  degrees of freedom
Residual deviance: 22.281  on 3  degrees of freedom
AIC: 42.436
Number of Fisher Scoring iterations: 4
```

***

```
              Out  A  B
AvgCost Stay
Hi      L         0 16
        S         0 36
Med     L         4 45
        S        14 61
Lo      L         0  7
        S        10 12

> S=factor(rep(c("Hi","Med","Lo"),c(2,2,2)))
> SAChi=(S=="Hi")
> SACmed=(S=="Med")
> B=factor(rep(c("L","S"),3))
> Bstay=(B=="L")
> y=c(16,36,45,61,7,12)
> n=c(16,36,45,61,7,12)+c(0,0,4,14,0,10)
> count=cbind(y,n-y)
> result=glm(count~Bstay+SAChi+SACmed, family=binomial("logit"))
> summary(result)

Call:
glm(formula = count ~ Bstay + SAChi + SACmed, family = binomial("logit"))
```

©2014 Q.H. Vuong

```
Deviance Residuals:
       1          2          3          4          5          6  
 0.00004    0.00012   -0.59704    0.30410    1.50469   -0.45122  

Coefficients:
             Estimate Std. Error z value Pr(>|z|)  
(Intercept)    0.3762     0.4100   0.918   0.3588  
BstayTRUE      1.3637     0.5757   2.369   0.0178 *
SAChiTRUE     21.9924  6896.4533   0.003   0.9975  
SACmedTRUE     1.0063     0.4791   2.100   0.0357 *
---
Signif. codes:  0 '***' 0.001 '**' 0.01 '*' 0.05 '.' 0.1 ' ' 1

(Dispersion parameter for binomial family taken to be 1)

    Null deviance: 33.2458  on 5  degrees of freedom
Residual deviance:  2.9166  on 2  degrees of freedom
AIC: 21.938

Number of Fisher Scoring iterations: 19

> result=glm(count~Bstay+SACmed, family=binomial("logit"))
> summary(result)

Call:
glm(formula = count ~ Bstay + SACmed, family = binomial("logit"))
Deviance Residuals:
      1        2        3        4        5        6  
 1.2126   3.4774  -0.5893   0.3006   0.8021  -3.3245  
Coefficients:
            Estimate Std. Error z value Pr(>|z|)    
(Intercept)   1.6989     0.3478   4.885 1.03e-06 ***
BstayTRUE     1.3582     0.5647   2.405   0.0162 *  
SACmedTRUE   -0.3154     0.4312  -0.731   0.4646    
---
Signif. codes:  0 '***' 0.001 '**' 0.01 '*' 0.05 '.' 0.1 ' ' 1

(Dispersion parameter for binomial family taken to be 1)
    Null deviance: 33.246  on 5  degrees of freedom
Residual deviance: 25.696  on 3  degrees of freedom
AIC: 42.717 - Number of Fisher Scoring iterations: 5
```
